# Novel Convenient Approach to the Solid-Phase Synthesis of Oligonucleotide Conjugates

**DOI:** 10.3390/molecules24234266

**Published:** 2019-11-22

**Authors:** Mariya I. Meschaninova, Darya S. Novopashina, Olga A. Semikolenova, Vladimir N. Silnikov, Alya G. Venyaminova

**Affiliations:** 1 Institute of Chemical Biology and Fundamental Medicine SB RAS, Lavrentiev ave.8, Novosibirsk 630090, Russia; mesch@niboch.nsc.ru (M.I.M.); silnik@niboch.nsc.ru (V.N.S.); ven@niboch.nsc.ru (A.G.V.); 2 Department of Natural Sciences, Novosibirsk State University, Pirogova str.2, Novosibirsk 630090, Russia; o.semikolenova@g.nsu.ru

**Keywords:** oligonucleotide conjugates, solid-phase 5′-functionalization, *N*,*N*′-disuccinimidyl carbonate (DSC), amino ligands, boron clusters

## Abstract

A novel and convenient approach for the solid-phase 5′-functionalization of oligonucleotides is proposed in this article. The approach is based on the activation of free 5′-hydroxyl of polymer support-bound protected oligonucleotides by *N*,*N*′-disuccinimidyl carbonate followed by interaction with amino-containing ligands. Novel amino-containing derivatives of *closo*-dodecaborate, estrone, cholesterol, and α-tocopherol were specially prepared. A wide range of oligonucleotide conjugates bearing *closo*-dodecaborate, short peptide, pyrene, lipophilic residues (cholesterol, α-tocopherol, folate, estrone), aliphatic diamines, and propargylamine were synthesized and characterized to demonstrate the versatility of the approach. The developed method is suitable for the conjugate synthesis of oligonucleotides of different types (ribo-, deoxyribo-, 2′-*O*-methylribo-, and others).

## 1. Introduction

Conjugates of oligonucleotides with various types of ligands combine the sequence-specific binding ability of oligonucleotides to nucleic acids with the functionality of ligands. A large variety of functional ligands, like peptides, vitamins, hormones, growth factors, enzymes, carbohydrates, lipophilic molecules, aptamers, polymers, fluorescent dyes, spin labels, metal complexes, nanoparticles, and solid surfaces, have been conjugated to DNA, RNA, or their analogs. The oligonucleotide conjugates attract particular attention as therapeutics for the treatment of cancer, genetic and viral diseases, and as imaging and diagnostic tools in biomedicine (see, e.g., [1,2,3,4,5,6,7,8,9,10,11,12]). They also find broad application in other fields such as the development of biohybrid nanomaterials and agents for biocatalysis, design of biomimetics, template-directed biosynthetic chemistry, magnetic and (opto)-electronic nanomaterial design (see, e.g., [13,14,15]). Taking into account the vast diversity of ligands and research tasks, the development of convenient and efficient approaches to the synthesis of oligonucleotide conjugates remains a significant challenge.

Ligands may be introduced at various positions of the molecule (5′- or 3′-terminus, sugar, phosphodiester backbone, or nucleobases). The synthetic strategies for oligonucleotide conjugates fall into two main categories: solution and solid-phase conjugation, each with its own benefits and drawbacks. Solid-phase synthesis of functionalized oligonucleotides most often relies on commercially available automated DNA/RNA synthesizers. Terminal functionalization of oligonucleotides or the site-specific introduction of modified nucleotides and non-nucleotidic monomers can be easily integrated into the automated synthesis protocol based on phosphoramidite or H-phosphonate chemistry. For example, in our previous works, we developed an approach for solid-phase synthesis of 5′-modified RNA with the use of using different H-phosphonates of lipophilic molecules [16,17,18]. Alternatively, post-synthetic modification of oligonucleotides may be performed on the solid support outside of the synthesizer. In this case, one can perform the functionalization not only through phosphoramidite/H-phosphonate chemistry, but also by other approaches, including Michael addition [19], or Cu(I)-catalyzed azide-alkyne Huisgen cycloaddition (see, e.g., [19,20]).

Two simple homobifunctional reagents have been routinely used in organic and bioorganic chemistry to activate the amino and hydroxyl moieties, 1,1′-carbonyldiimidazole (CDI) and *N*,*N*′-disuccinimidyl carbonate (DSC) [21]. The reactivity of the *N*-succinimidyl carbonate formed during DSC activation is much higher than that of imidazole carbamate, the active species in CDI activation. The carbamate linkage formed after the coupling of succinimidyl carbonate to amine-containing ligands is chemically stable [22]. In the context of nucleic acid chemistry, the activation of 5′-hydroxyl by CDI for the synthesis of 5′-aminoalkyl oligodeoxynucleotides was described earlier [23,24], but no examples of using DSC have been reported to date.

In the present work, we have developed a new approach to the solid-phase 5′-modification of oligonucleotides using *N*,*N*′-disuccinimidyl carbonate. The approach is based on the activation of free 5′-hydroxyl of protected oligonucleotides (ribo-, deoxyribo- or 2′-*O*-methylribo-) by DSC followed by the interaction with amino containing ligands of different chemical nature.

## 2. Results

*N,N*′-Disuccinimidyl carbonate is widely used for the preparation of modified surfaces as well as for the introduction of polyethylene glycol (PEG) to the definite sites of proteins or nucleic acids (see, e.g., [25,26,27]). The advantages of DSC as compared to CDI include longer shelf life, a wide range of suitable solvents, and higher reactivity of the activated hydroxyl. The activation of 5′-amino group of solid-phase-attached oligodeoxyribonucleotide by DSC was successfully used for peptide conjugates preparation in previous works [28,29]. However, much to our surprise, DSC has not yet been employed for the synthesis of oligonucleotides conjugated through the activation of 5′-hydroxyl of polymer support-bound oligonucleotides.

**Scheme 1:**
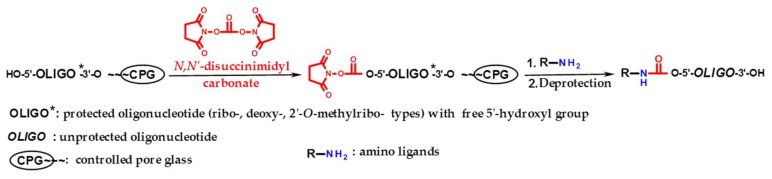
The general scheme of the solid-phase 5′-functionalization of oligonucleotides (ribo-, 2′-*O*-methylribo-, and deoxyribo-). R–NH_2_ structures see Supplementary Table S1.

The general scheme of the proposed solid-phase 5′-functionalization of oligonucleotides includes two stages (Scheme 1): 1) the activation of 5′-hydroxyl of protected oligonucleotide by DSC with the formation of the intermediate 5′-succinimidyl carbonate, and 2) the interaction of this intermediate with an amino ligand followed by standard deprotection of resulted oligonucleotide conjugate. 

Amine-containing ligands of various structures were coupled with activated 5′-hydroxyl group of oligonucleotides (Supplementary, Table S1). We used a range of amines of *closo*-dodecaborate and lipophilic molecules (cholesterol, folic acid, α-tocopherol, estrone), manufactured by us in our laboratory, as well as a series of commercially available amino ligands (pyrenemethylamine, oleylamine, trileucine, propargylamine, aliphatic diamines, 3-aminopropan-1-ol, 12-aminododecan-1-ol).

**Scheme 2:**
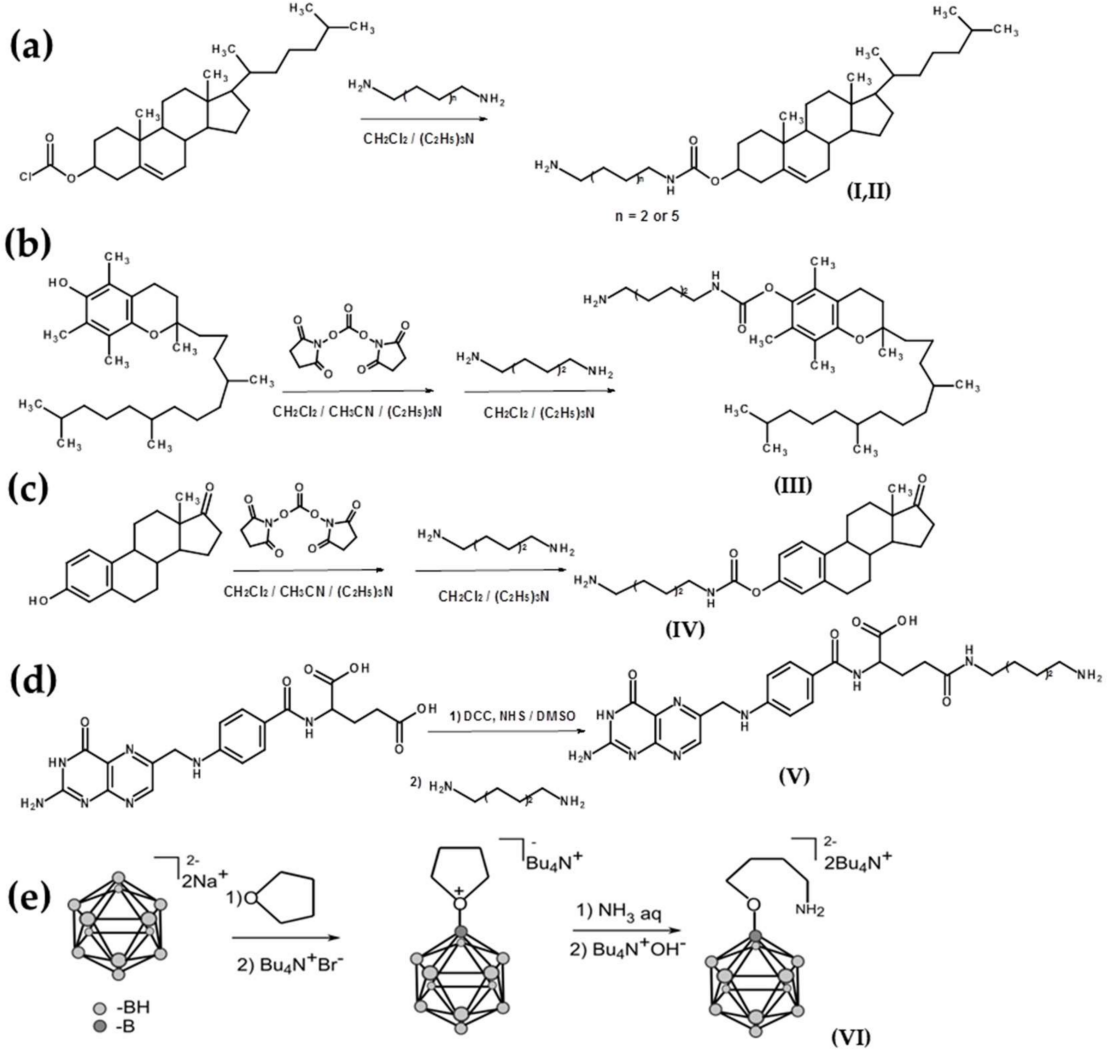
Schemes of the synthesis of amino-modified ligands: (**a**) cholesterols (**I, II**), (**b**) α-tocopherol (**III**), (**c**) estrone (**IV**), (**d**) γ-amino-modified folate (**V**), and (**e**) aminotetramethylene oxonium derivative of *closo*-dodecaborate (**VI**). NHS, *N*-hydroxysuccinimide; DCC, *N*,*N*′-dicyclohexylcarbodiimide.

The synthesis of amino-modified cholesterols (**I, II**) was performed by analogy with previous works [30] (Scheme 2a). Cholesteryl chloroformate was introduced to the reaction with an excess of diamine (hexamethylenediamine or dodecamethylenediamine) in CH_2_Cl_2_, then the solution was washed by water to remove the unreacted diamine, and amino-modified cholesterols were isolated by column chromatography. For the preparation of amino-modified α-tocopherol (**III**) and estrone (**IV**), the hydroxy groups were activated by DSC followed by interaction with hexamethylenediamine by analogy with previous work [31,32] (Scheme 2b,c) and isolated by column chromatography. The γ-carboxylic group of folic acid was activated by *N*,*N*′-dicyclohexylcarbodiimide (DCC) in the presence of *N*-hydroxysuccinimide during 16 h at room temperature (r.t.). Activated folic acid was reacted with an excess of hexamethylenediamine by analogy with previous work [33] (Scheme 2d), and γ-amino-modified folate (**V**) was isolated by column chromatography.

An aminotetramethylene oxonium derivative of *closo*-dodecaborate (**VI**) was prepared by the treatment of *closo*-dodecaborate anion with BF_3_∙Et_2_O in tetrahydrofuran and subsequent interaction with ammonia (NH_3 aq_) (Scheme 2e) [34,35]. For further information on processing, see Materials and Methods.

The structures of all amino-modified ligands were confirmed by NMR (see Materials and Methods for details).

**Scheme 3:**
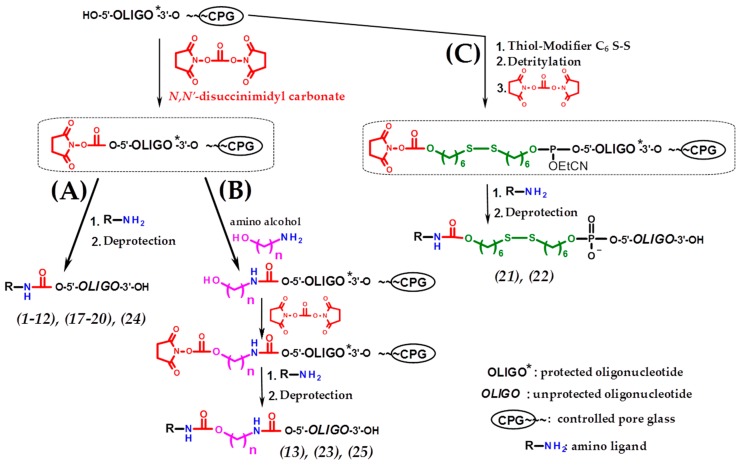
Solid-phase synthesis of 5′-conjugates of oligonucleotides (Table 1) based on DSC activation. (**A**) Direct synthesis of 5′-functionalized oligonucleotides based on DSC activation; (**B**) introduction of prolonged linkers by carrying out several successive activation/addition solid-phase reactions through DSC; (**C**) the use of non-nucleotidic biolabile linker with subsequent activation of hydroxyl of this linker by DSC and attachment of the amino ligand.

**Figure 1:**
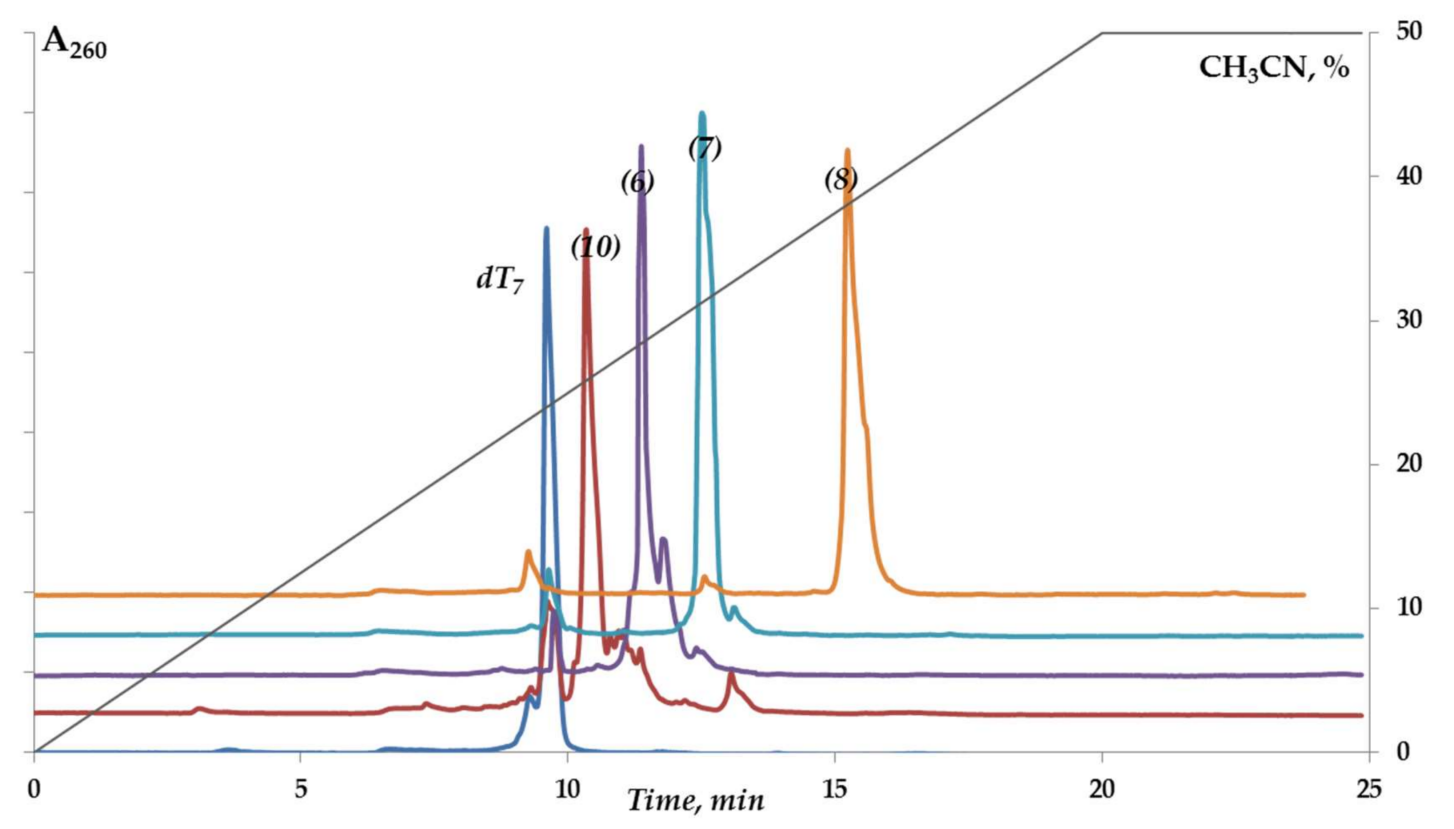
RP HPLC analysis of reaction mixtures upon conjugation of dT_7_ with different amino ligands (Table 1). See Materials and Methods for details.

We demonstrated the feasibility of our solid-phase approach for the synthesis of 5′-functionalized oligonucleotides based on DSC activation using the wide range of conjugates of the model oligodeoxyribonucleotide dT_7_ (***1***–***12***) (Table 1, Scheme 3A). The optimal solvent for solid-phase attachment of each particular amino ligand was thoroughly selected (Supplementary Table S1). The degrees of conversion of dT_7_ to the conjugates were about 80–95% (Figure 1, Supplementary Figure S1). The retention times of dT_7_ conjugates in reversed-phase high-performance liquid chromatography (RP HPLC) varied depending on the nature of the ligand. Conjugates with cholesterol (***1***,***2***), pyrene (***8***), and dodecamethylenediamine (***11***) had the highest values for the retention time (Table 1).

The conjugates of model dT_7_ and 20-mer hetero-oligodeoxyribonucleotide with an aminotetramethylene oxonium derivative of *closo*-dodecaborate (***9, 17***) were prepared using the same DSC-based approach (Table 1, Scheme 3A).

Conjugates of anti-mdr1 siRNA sense strand [36,37] with trileucine (***19***) and cholesteryl-6-aminohexylcarbamate (***20***) were also successfully synthesized in optimal conditions (Table 1, Scheme 3A). Moreover, the proposed approach allows, when necessary, to lengthen the spacer between the oligonucleotide and the ligand by several successive activation/addition solid-phase reactions through DSC. This possibility was demonstrated by using amino alcohols of various structures to obtain the cognate conjugates (***13***,***23***,***25***) (Table 1, Scheme 3B).

We have also demonstrated the possibility of applying the DSC-based approach for the activation of hydroxyl of non-nucleotidic biolabile linkers introduced upon oligonucleotide solid-phase synthesis. For this purpose, commercially available disulfide-containing phosphoramidite (Thiol-Modifier) was used for the synthesis of 5′-S-S-modified anti-mdr1 siRNA sense strand with subsequent activation of hydroxyl of Thiol-Modifier by DSC and coupling with trileucine (conjugate (***21***)) or cholesteryl-6-aminohexylcarbamate (conjugate (***22***)) (Table 1, Scheme 3C).

## 3. Discussion

In the present work, we have proposed and validated the novel method of solid-phase synthesis of oligonucleotide conjugates with different amino ligands. The approach is based on the activation of the free 5′-hydroxyl of protected polymer-bound oligonucleotide obtained by an automated oligonucleotide synthesis with *N*,*N′*-disuccinimidyl carbonate (DSC), followed by the interaction with amino-containing ligands and standard deprotection steps (Scheme 1).

**Figure 2:**
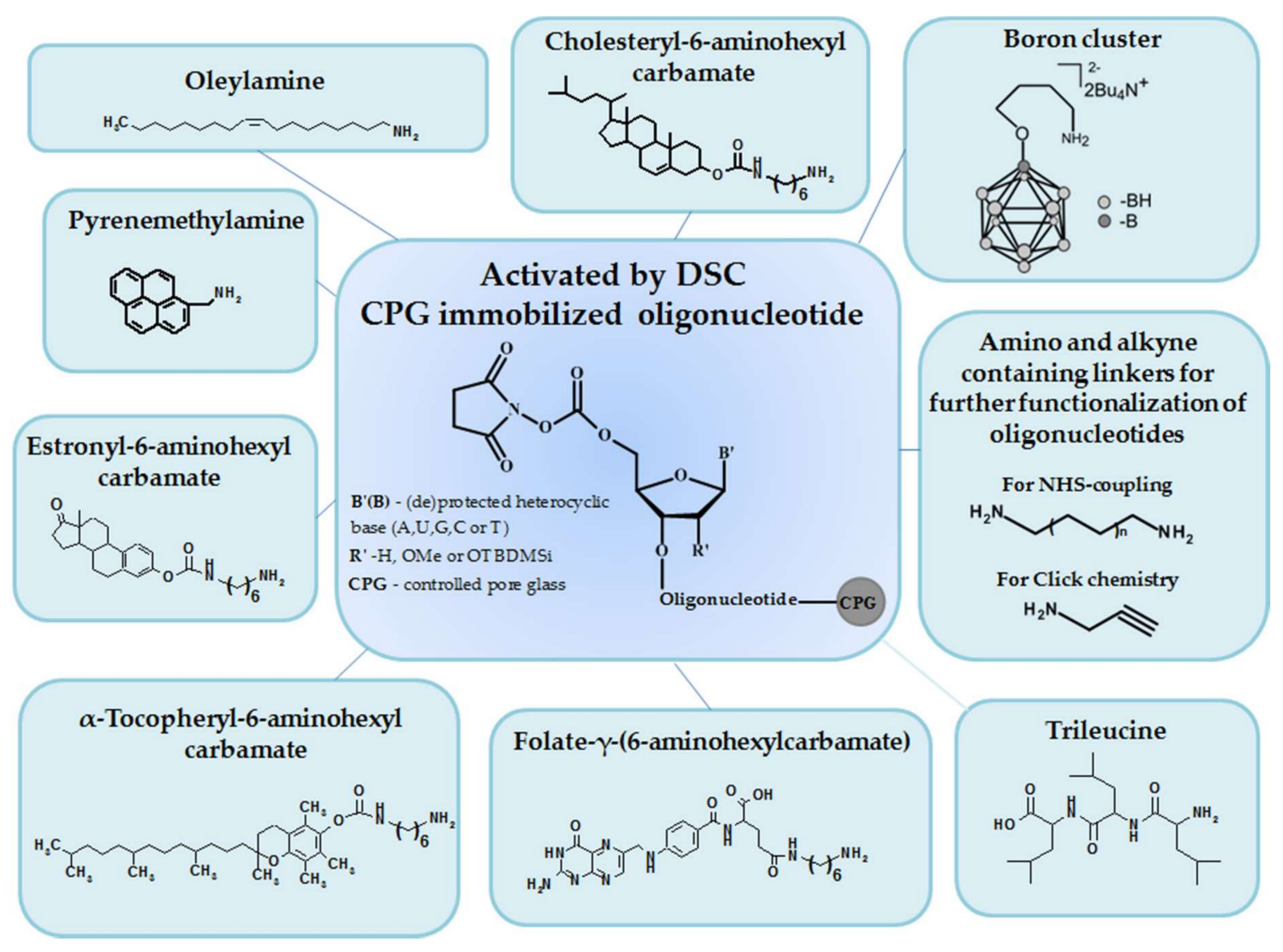
Possible applications of the proposed solid-phase approach for the synthesis of 5′-conjugates of oligonucleotides.

We demonstrated the proof of principle for our approach on a wide range of amino-modified bioactive and functional ligands that are interesting from a practical point of view (lipophilic molecules, short peptides, fluorophores, etc.) (Table 1, Figure 2).

For example, we employed this approach to conjugate oligonucleotides with different types of amino ligands which facilitate the cell penetration, such as lipophilic molecules (cholesterol, estrone, oleylamine, α-tocopherol, folic acid) and short peptides (trileucine) (Table 1, Scheme 3A, (***1***–***7***), (***18***–***20***)). Of note, the developed DSC-based solid-phase approach is especially convenient for the synthesis of oligonucleotide conjugates bearing highly hydrophobic residues since there is no need to seek the solvents that accommodate both extreme lipophiles and hydrophilic oligonucleotides.

The attachment of trileucine improves the intracellular delivery and endosomal escape of oligonucleotides [38,39]. In the case of peptide attachment, it is necessary to take into account the possibility of reaction with side-chain amino groups and peptide damage upon exposure to the harsh basic deprotection conditions used for removing the protective groups of the oligonucleotides. Additionally, peptides should be stable during the 2′-deprotection in the case of peptide conjugates of oligoribonucleotides. Here, we used trileucine since it is stable upon basic treatment and 2′-deprotection of oligoribonucleotides, and does not contain side-chain amino groups.

The key point upon the design of oligonucleotide therapeutics and probes is the proper choice of linker between the oligonucleotide and functional moiety, such as delivery agent, fluorophore, or other reporter groups [8,18,40,41]. Our method permits varying the size and structure of the linker to optimize its length and stability for the particular task. To illustrate this possibility, we synthesized a series of 5′-modified sense strands of anti-mdr1 siRNA bearing trileucine or cholesterol attached either directly to the RNA (conjugates (***19***, ***20***), Scheme 3A) or via stable and biolabile linkers (conjugates (***23***) and (***21***, ***22***), respectively, Scheme 3B,C). The DSC-based method makes it possible to perform several successive activation/addition solid-phase reactions with different amino ligands without isolation of intermediate derivatives. For example, we increased the length of stable linker within the 5′-cholesterol conjugate of the sense strand of siRNA (***20***) by the sequential attachment of 12-aminododecan-1-ol to the 5′-hydroxyl of RNA through DSC activation and the reaction with cholesteryl-6-aminocarbamate after the additional DSC activation of the linker’s hydroxyl (conjugate (***23***), Scheme 3B). A biolabile disulfide-containing linker (***LSSL***) was incorporated into siRNA strand at the final stage of solid-phase oligonucleotide synthesis through corresponding non-nucleotidic phosphoramidite with subsequent activation of the hydroxyl group of the linker by DSC and interaction with trileucine or cholesteryl-6-aminocarbamate, giving conjugate (***21***) or conjugate (***22***) respectively (Scheme 3C).

The developed method for 5′-conjugation was also successfully employed for the synthesis of fluorescently labeled oligonucleotides as hybridization probes for nucleic acid detection or visualization. A hydrophobic fluorophore pyrene was attached to model dT_7_ and oligo(2′-*O*-methylribonucleotide) either directly as pyrenemethylamine (Scheme 3A for (***8***, ***24***)) or through the introduction of aminopropanol as a linker (Scheme 3B for (***13***, ***25***)).

The incorporation of boron clusters into nucleic acids appears promising for the creation of new boron delivery agents for boron neutron capture therapy (BNCT) capable of specific interaction with nucleic acids inside the cells [42]. Several methods were developed for modification of oligonucleotides with boron clusters attached to internal positions of an oligonucleotide chain, such as sugar residues, nucleobases, or phosphorus atoms of the internucleotide linkages [14,43]. The reported methods for the synthesis of oligonucleotides modified with borane clusters include a post-synthetic “click”-chemistry approach based on Huisgen 1,3-dipolar cycloaddition (see, e.g., [44]) and an H-phosphonate or phosphoramidite solid-phase approach using modified nucleotide monomers (see, e.g., [45]). The DSC-based method developed in this study represents an efficient alternative to currently used approaches and permits attachment of a boron cluster to the oligonucleotide terminus. The attachment of amino-modified *closo*-dodecaborates to the 5′-terminus of oligonucleotides gives the cognate boron-containing conjugates (***9***, ***17***) (Scheme 3A). It can be expected that the introduction of *closo*-dodecaborates to the 5′-terminus would not significantly disturb the complementary interactions of modified oligonucleotide with nucleic acid targets and their secondary structure, in contrast to the internal modifications mentioned above.

5′-Amino- and 5′-alkyne-modified oligonucleotides are a well-known platform for the preparation of different conjugates. The proposed method is quite versatile for obtaining 5′-amino- and 5′-alkyne-modified oligonucleotides using diamines or aminoalkynes of different length and structure. This possibility was validated through the model synthesis of aliphatic diamine-modified oligonucleotides (***10***, ***11***) or propargylamine-modified oligonucleotide (***12***) in the presence of DSC (Scheme 3A) followed by well-known reactions with *N*-(2-hydroxyethyl)phenazinium chloride or Cy3 azide (Supplementary, Figures S2 and S3, respectively).

Therefore, the developed approach is highly versatile in relation both to oligonucleotide nature and functional ligand type, and can be applied to the synthesis of functional RNAs, DNAs, and their analogs to be decorated by ligands of interest depending on the particular research task.

## 4. Materials and Methods

### 4.1. Chemicals and Reagents

A controlled pore glass support (CPG) derivatized with 2′-*O*-TBDMS-G, 2′-*O*-methyl-G, deoxycytosine (dC) or deoxythymidine (dT), 5′,*N*-protected 2′-*O*-methylribo- (A, C, G or U), 2′-*O*-TBDMS-ribo (A, C, G or U) and deoxyribo (dA, dC, dG or dT,) phosphoramidites, 1-*O*-dimethoxytrityl-hexyl-disulfide,1′-[(2-cyanoethyl)-(*N*,*N*-diisopropyl)]-phosphoramidite (Thiol-Modifier C6 S–S) were purchased from Glen Research Inc (Sterling, VA, USA). (Pyrene-1-yl-methyl)amine hydrochloride, *N,N*-diisopropylethylamine (DIPEA), trileucine, α-tocopherol, propargylamine, hexamethylenediamine, were purchased from Sigma-Aldrich (St. Louis, MO, USA), *N*,*N*′-disuccinimidyl carbonate (DSC), cholesterol chloroformate, folic acid, oleylamine were purchased from Acros Organics (Geel, Belgium), Cy3-azide, 10 mM Cu(II)-TBTA Stock in 55% DMSO, ascorbic acid were purchased from Lumiprobe (Russia), dodecamethylenediamine was from Fluka (St. Louis, MO, USA), 12-amino-1-dodecanol was from TCI Chemicals (India), and sodium dodecaborate Na_2_[B_12_H_12_] was from AviaBor (Dzerzhinsk, Russia). *N*-(2-hydroxyethyl)phenazinium chloride was obtained in ICBFM SB RAS as described in previous work [44]. All solvents (THF, DMSO, CH_3_CN (various vendors)) were dried by 3 Å molecular sieves or by distillation and stored over CaH_2_. Kieselgel F254 thin-layer chromatography plates were purchased from Merck (Kenilworth, NJ, USA).

### 4.2. Physical Measurements

^1^H-NMR spectra of the compounds were measured with CDCl_3_, *d*-DMSO, or acetone-d6 as solvents using AVANCE III 400 NMR spectrometer (Bruker Corporation, Billerica, MA, USA).

Mass spectra were recorded using a MALDI-TOF Autoflex Speed mass spectrometer (Bruker Daltonics, Billerica, MA, USA) or Agilent G6410A LC-MS/MS Instrument (Agilent Technologies, CIIIA).

The optical densities of the solutions of oligonucleotide conjugates were measured using a NanoDrop 1000 spectrophotometer (Thermo Fisher Scientific, Waltham, MA, USA).

### 4.3. Synthesis of Polymer-Bound Oligonucleotides

Oligodeoxyribonucleotides, oligo(2′-*O*-methylribonucleotide), oligo(ribo-/2′-*O*-methylribo)nucleotides and their 5′-S-S-derivative were synthesized on an automatic ASM-800 synthesizer at 0.4 mmol scale using solid-phase phosphoramidite synthesis protocols [46] optimized for the instrument, with a 3 min coupling step for deoxy-phosphoramidites (0.05 M in CH_3_CN), 10 min coupling step for 2′-*O*-TBDMS-protected phosphoramidites (0.1 M in CH_3_CN), 6 min coupling step for 2′-*O*-methyl-phosphoramidites (0.05 M in CH_3_CN), 10 min coupling step for Thiol-Modifier C6 S–S phosphoramidites (0.1 M in CH_3_CN) and 5-ethylthio-1*H*-tetrazole (0.25 M in CH_3_CN) as an activating agent. A mixture of acetic anhydride with 2,6-lutidine in THF and *N*-methylimidazole in THF were utilized as capping reagents. The oxidizing agent was 0.02 M iodine in pyridine/water/THF (1/9/90). Dichloroacetic acid (3%) in CH_2_Cl_2_ was used as a detritylating reagent. Polymer-bound DMTr-off oligonucleotides were used for solid-phase conjugation.

### 4.4. Preparation of Amino Compounds 

Cholesteryl-6-aminohexylcarbamate (**I**) and cholesteryl-12-aminododecanylcarbamate (**II**) were obtained by analogy with previous work [30]. Diamine (8 mmol) and triethylamine (30 μL, 2 μmol) were dissolved in 7.5 mL of dry CH_2_Cl_2_, and the mixture was stirred at 0 °C. The solution of cholesteryl chloroformate (2 mmol) in CH_2_Cl_2_ (3 mL) was added dropwise to the reaction mixture and stirred overnight at r.t. The reaction was monitored by TLC (10% EtOH in CH_2_Cl_2_). After that, the mixture was filtered, the filtrate was washed with water 5 times, dried over anhydrous Na_2_SO_4_, and evaporated in vacuum to dryness. The products were isolated by column chromatography and evaporated to dryness. The yields were 72 and 45%, respectively.

Cholesteryl-6-aminohexylcarbamate (**I**). ^1^H-NMR (400 MHz, CDCl_3_, ppm): 5.34 (s, 1H, alkenyl), 4.60 (s, 1H, COONH), 4.47 (m, 1H, oxycyclohexyl), 3.24–3.26 (q, 2H, NHCH_2_), 2.75–2.86 (t, 2H, NH_2_CH_2_), 1.22 (m, 10H, (CH_2_)_4_), 0.64–1.63 (m, 45H, cholesteryl protons).

Cholesteryl-12-aminododecanylcarbamate (**II**). ^1^H-NMR (400 MHz, CDCl_3_, ppm): 5.4 (s, 1H, alkenyl), 4.88 (s, 1H, COONH), 4.51 (m, 1H, oxycyclohexyl), 3.35 (q, 2H, NHCH_2_), 2.35 (t, 2H, NH_2_CH_2_), 1.02 (m, 4H, –(CH_2_)_4_–), 0.69–1.43 (m, 45H, cholesteryl protons).

α-Tocopheryl-6-aminohexylcarbamate (**III**) and estronyl-6-aminohexylcarbamate (**IV**) were prepared as in previous works [31,32]. α-Tocopherol or estrone (1 mmol) was dissolved in dry CH_2_Cl_2_ (3 mL) with subsequent adding of triethylamine (0.42 mL, 3 mmol) and solution of *N*,*N*′-disuccinimidyl carbonate (0.38 g, 1.5 mmol) in 3 mL of CH_3_CN. The reaction mixture was stirred for 24 h at r.t. After that, the reaction mixture was washed by the saturated water solution of NaHCO_3_, dried over anhydrous Na_2_SO_4_, and evaporated in vacuum to dryness. These intermediates were used in the next step without any additional purification. Hexamethylenediamine (0.35 g, 3 mmol) was suspended in 3 mL of CH_2_Cl_2_. Succinimidyl carbonates (1 mmol) obtained on the previous step were dissolved in 3 mL of CH_2_Cl_2_ and dropwise added to the diamine solution at 0 °C. Reaction mixtures were stirred during 16 h at r.t., evaporated in vacuum to dryness (oil), and dissolved in 50 mL of CH_2_Cl_2_, washed twice by saturated water solution of NaHCO_3_, then with water 5 times, dried over anhydrous Na_2_SO_4_, and evaporated in vacuum to dryness. The products were isolated by column chromatography. The yields were 80 and 65%, respectively.

α-Tocopheryl-6-aminohexylcarbamate (**III**). ^1^H-NMR (400 MHz, CDCl_3_, ppm): 5.09 (t, 1H, –NH–), 3.27 (dd, 2H, –CH_2_–NH–), 2.58 (t, 2H, 4,4-H, α-tocopherol), 2.37 (t, 2H, –CH_2_–COO), 2.08 (s, 3H, –CH_3_, α-tocopherol), 2.06 (s, 3H, –CH_3_, α-tocopherol), 2.02 (s, 3H, –CH_3_, α-tocopherol).

Estronyl-6-aminohexylcarbamate (**IV**). ^1^H-NMR (400 MHz, CDCl_3_, ppm): 7,15 (d, 1H, estrone), 6,8 (d, 1H, estrone), 6,72 (d, 1H, estrone), 3.25 (t, 1H, –NH–), 3.13 (dd, 2H, –CH_2_–NH–), 3,04 (t, 2H, estrone), 1.25 (m, 4H, –(CH_2_)_4_–), 1.23 (s, 3H, –CH_3_, estrone).

Folate-6-aminohexylcarbamate (**V**) was obtained by analogy with previous work [33]. Folic acid (320 mg, 0.62 mmol) was dissolved in 12 mL of DMSO at 50 °C by shaking for 30 min. After that, *N*-hydroxysuccinimide (154 mg, 1.34 mmol) and DCC (276 mg, 1.34 mmol) were added. The reaction mixture was stirred for 16 h at r.t., then the urea precipitate was filtered off. The filtrate was added dropwise to the solution of hexamethylenediamine (3.1 mmol) and triethylamine (1.34 mmol) in DMSO (5 mL). The mixture was stirred for 18 h, then added to 20% acetone in diethyl ether. The thin yellow precipitate was carefully centrifuged, washed four times with acetone and twice with diethyl ether, and dried in vacuum. The yield was 68%. ^1^H-NMR (400 MHz, d-DMSO, ppm) 8.63 (s, 1H, pterin), 7.98 (m, 1H), 7.56–7.67 (m, 2H, aromatic, 6.93 (m, 3H), 6.57–6.63 (m, 2H, aromatic), 4.48 (bs, 2H, benzylic), 4.20–4.30 (m, 1H), 2.97–3.01 (m, 6H, –CH_2_–), 2.72–2.76 (m, 2H, –CH_2_–), 2.33 (m, 4H, glutamic moiety).

Bis-tetrabutylammonium-(4-aminobuthoxy)-undecahydro-*closo*-dodecaborate (**VI**) was obtained according to [34,35]. BF_3_xEt_2_O (0.7 mL, 0.78 g, 5.5 mmol) was added to a stirred solution of 0.95 g (5.0 mmol) Na_2_(B_12_H_12_) in 25 mL of dry THF and the reaction mixture was stirred for 12 h at r.t. The reaction mixture was filtered, and the solvent was distilled off from the filtrate. The residue was dissolved in 70 mL water and treated with a solution of 3.22 g (10.0 mmol) Bu_4_NBr in water. The precipitate formed was filtered off, dried in air, and recrystallized from methanol, giving the tetrahydrofurane-*closo*-dodecaborate complex as a crystalline product in 84% yield. ^1^H-NMR (400 MHz, CDCl_3_, ppm): 4.52 (4H, dt, –O(CH_2_CH_2_)_2_), 3.21 (8H, m, Bu_4_N^+^–), 2.15 (4H, dt, –O(CH_2_CH_2_)_2_), 1.62 (8H, m, Bu_4_N^+^–), 1.44 (8H, q, Bu_4_N^+^–), 0.98 (12H, t, Bu_4_N^+^–).

A solution of 25% NH_3_ aq. (4 mL, 105 mmol) was added to tetrahydrofurane-*closo*-dodecaborate complex (1 g, 2.2 mmol) in CH_3_CN (8 mL). The mixture was stirred at 50 °C for 11 h, cooled to room temperature and then concentrated under reduced pressure. The corresponding ring-opening product was obtained as a white solid.

The resulting product was suspended in MeOH and 10% (*w/w*) tetrabutylammonium hydroxide (TBAOH) in MeOH (6.73 mL, 2.54 mmol) was added. The mixture was stirred until the white solid was dissolved. After removing the solvent under reduced pressure, the residue was dissolved in CH_2_Cl_2_ and washed with water 3 times. The CH_2_Cl_2_ phase was dried over MgSO_4_ and concentrated to give amine as a white solid (1.4 g, 1.95 mmol, 89%). ^1^H-NMR (400 MHz, acetone-d6, ppm): 3.60 (2H, t, –CH_2_OB_12_); 3.42 (16H, m, N^+^CH_2_CH_2_CH_2_CH_3_); 3.08 (2H, m, –CH_2_NH_2_); 1.79 (20 H, m, OCH_2_CH_2_CH_2_CH_2_N and N^+^CH_2_CH_2_CH_2_CH_3_);1.43 (16H, m, N^+^CH_2_CH_2_CH_2_CH_3_); 0.97 (24H, t, N^+^CH_2_CH_2_CH_2_CH_3_).

### 4.5. Solid-Phase Synthesis of Oligonucleotide Conjugates

A) Synthesis of conjugates (***1–12***, ***17–22***, ***24***) (see Scheme 3A,C): *N*,*N*′-disuccinimidyl carbonate (6.5 mg, 25 μmol) in a mixture of CH_3_CN (270 μL) and DIPEA (30 μL) was added to the dried polymer-bound DMTr-off oligonucleotides or 5′-thiol-modified oligonucleotide (0.25–0.5 μmol). The mixture was stirred (1500 rpm) for 1 h at 37 °C. Then the solution was replaced by freshly prepared DSC solution of the same concentration and stirred for another 30 min. The procedure was repeated twice. After that, the solution was removed, and freshly prepared solution of amino ligand (12 μmol) in 200 μL mixture of an optimal solvent (THF, CH_3_CN, DMSO, see Table S1) and DIPEA (10%, *v/v*) was added and stirred at 37 °C. After 1 h, the second portion of the amino ligand solution with the same concentration was added to the polymer-bound oligonucleotide. The reaction mixture was stirred overnight at 37 °C. Then, the solution was removed, the polymer was washed twice by 300 μL of a suitable solvent (THF, CH_3_CN, DMSO), twice by 300 μL acetone, and dried in air.

B) Synthesis of conjugates (***13***, ***23***, ***25***) (see Scheme 3B): *N*,*N*′-disuccinimidyl carbonate (6.5 mg, 25 μmol) in the mixture of CH_3_CN (270 μL) and DIPEA (30 μL) was added to the dried polymer-bound DMTr-off oligonucleotide (0.25–0.5 μmol). The mixture was stirred (1500 rpm) for 1 h at 37 °C. Then, the solution was replaced by freshly prepared DSC solution with the same concentration and stirred for another 30 min. The procedure was repeated twice. After that, the solution was removed, and freshly prepared solution of amino alcohol (12 μmol) in 200 μL mixture of DMSO and DIPEA (10%, *v/v*) was added and stirred at 37 °C. After 1 h, the second portion of amino alcohol solution with the same concentration was added to the polymer-bound oligonucleotide. The reaction mixture was stirred overnight at 37 °C. The solution was removed, the polymer was washed twice by DMSO (300 μL), twice by acetone (300 μL), and dried in air. The subsequent introduction of pyrenemethylamine or cholesteryl-6-aminohexylcarbamate was fulfilled as described above (4.5.A).

### 4.6. Deprotection and Isolation of the Conjugates

The oligonucleotide conjugates were cleaved from the support and deprotected by 40% methylamine in the water at 65 °C for 15 min. 2′-*O*-TBDMS groups were removed upon treatment with a mixture of NMP/TEA·3HF/TEA (150/100/75) at 65 °C for 1.5 h. Deprotected conjugates were isolated by 12% denaturing polyacrylamide gel electrophoresis (PAGE), followed by elution from the gel with 0.3 M NaClO_4_ solution, desalted with Amicon Ultra 3K (Millipore, Burlington, MA, USA) or Sep-Pak C18 cartridge (Waters, Milford, MA, USA) and precipitated as sodium salts. The purified oligonucleotide conjugates were characterized by RP-HPLC and mass spectrometry (Table 1). Unmodified control oligonucleotides were deblocked in the same way.

### 4.7. Synthesis of N-(2-Hydroxyethyl)phenazinium Conjugates (**14**, **15**)

The *N*-(2-hydroxyethyl)phenazinium derivatives (***14***, ***15***) were obtained by analogy with previous work [47]. The solution of the *N*-(2-hydroxyethyl)phenazinium chloride (0.05 M, 80 μL) in 0.1 M Na_2_CO_3_ was added to the amino-modified oligonucleotides (***10***,***11***) (Na^+^ salts). The mixture was incubated for 10 min, precipitated with 2% solution of NaClO_4_ in acetone, washed by acetone, and dried in air. The reaction mixture was dissolved and analyzed using RP HPLC (Supplementary, Figure S2). The values for degree of oligonucleotide conversion to the conjugates were about 85–95% according to the HPLC data.

### 4.8. Synthesis of Cy3 Conjugate (**16**)

Triethylammonium acetate buffer (pH 7.0), 10 mM Cy3-azide in DMSO, 5 mM ascorbic acid solution in water and 10 mM Cu(II)-TBTA stock in 55% DMSO were added to the water solution of 5′-alkyne-modified oligonucleotide (***12***) (25 nmol) according to the protocol of click reagent supplier (Lumiprobe, Moscow, Russia). The reaction mixture was incubated at r.t. overnight. The oligonucleotide conjugate was precipitated with 2% NaClO_4_ in acetone and washed with acetone. The pellet was dried, dissolved in water, and analyzed by gel electrophoresis Supplementary Figure S3). The conversion of the oligonucleotide to the conjugate was almost quantitative according to the PAGE data.

### 4.9. RP HPLC Analysis of the Oligonucleotide Conjugates

Reversed-phase HPLC (RP-HPLC) analysis of the oligonucleotides and their conjugates was performed on an Alphachrom A-02 high performance liquid chromatograph (EcoNova, Novosibirsk, Russia) with the use of ProntoSil-120-5-C18 AQ (75 × 2.0 mm, 5.0 μm) column, applying a gradient elution from 0% to 50% (20 min) of CH_3_CN in 0.02 M triethylammonium acetate buffer, pH 7.0 at a flow rate 100 μL per min, and detection at 260 and 280 nm (345 nm for the pyrene conjugate).

## 5. Conclusions

Covalent conjugation of nucleic acids with different types of ligands is a powerful strategy to improve their chemical or biological properties or to confer them a complex of new characteristics during the development of new tools for synthetic biology, biomedicine, or bionanotechnology. Here, we present a novel convenient approach to the solid-phase 5′-functionalization of oligonucleotides, which relies on the activation of 5′-hydroxyl of protected polymer-bound oligonucleotide by *N*,*N’*-disuccinimidyl carbonate and subsequent interaction with an amino ligand. The versatility of the method was validated by conjugation of oligonucleotides with ligands of various structure and functionality, including lipophilic molecules, peptides, fluorophores, boron clusters, and diamines, introduced via short or long linkers, both stable or biolabile. The developed approach was successfully employed for 5′-functionalization of DNA, RNA, and 2′-*O*-methyl RNA, and it may be suggested that it is also suitable for conjugates of other types of NA analogs.

Taken together, our results confirm the useful contribution of this method in the design of the diversity of functional NA conjugates.

## Figures and Tables

**Table 1:** Synthesized conjugates of oligonucleotides and their characteristics.

N	Conjugate, 5′–3′	RP HPLC Retention Time, min^1^	Molecular Weight		Yield, %^3^
			Calculated	Experimental^2^	
***1***	*Chol**L_6_**–*NH–C(O)–d(TTTTTTT)	13.1 (+3.5)	2623.2	2624.7	10.5
***2***	*Chol**L_12_**–*NH–C(O)–d(TTTTTTT)	18.2 (+8.6)	2707.0	2708.6	3.2
***3***	*Oleyl–*NH–C(O)–d(TTTTTTT)	12.2 (+2.6)	2361.9	2361.3	18.2
***4***	*Est**L**_6_–*NH–C(O)–d(TTTTTTT)	12.3 (+2.7)	2506.9	2507.6	3.4
***5***	*Toc**L_6_**–*NH–C(O)–d(TTTTTTT)	11.1 (+1.5)	2667.3	2668.2	2.8
***6***	*Fol**L_6_**–*NH–C(O)–d(TTTTTTT)	11.8 (+2.2)	2677.9	2676.2	16.1
***7***	*(Leu)**_3_**–*NH–C(O)–d(TTTTTTT)	12.5 (+2.9)	2451.9	2352.1	25.4
***8***	*Pyr–*NH–C(O)–d(TTTTTTT)	15.9 (+6.3)	2325.7	2325.8	18.6
***9***	*c–B_12_–*NH–C(O)–d(TTTTTTT)	11.2 (+1.6)	2303.0	2301.6	7.1
***10***	*NH_2_**L_6_**–*NH–C(O)–d(TTTTTTT)	10.4 (+0.8)	2210.6	2210.4	16.3
***11***	*NH_2_**L_12_**–*NH–C(O)–d(TTTTTTT)	14.3 (+4.7)	2294.8	2293.4	15.1
***12***	*CH≡C–*CH_2_*–*NH–C(O)–d(TTTTTTT)	10.8 (+1.2)	2149.5	2149.5	17.2
***13***	*Pyr*–NH–C(O)–***L_3_****–*NH–C(O)–d(TTTTTTT)	17.0 (+7.4)	2427.8	2426.1	7.5
***14***	*Phn–NH**L_6_**–*NH–C(O)–d(TTTTTTT)	13.2(+3.6)	n.d.^5^	n.d.^5^	n.d.^5^
***15***	*Phn–NH**L_12_**–*NH–C(O)–d(TTTTTTT)	17.2(+7.6)	n.d.^5^	n.d.^5^	n.d.^5^
***16***	*Cy3**L**–*CH_2_–NH–C(O)–d(TTTTTTT)	n/a^4^	n.d.^5^	n.d.^5^	n.d.^5^
***17***	*c–B_12_–*NH–C(O)–d(ATACGTTAACGATCCTTCAC)	n/a^4^	6281.2	6282.0	5.1
***18***	*Oleyl–*NH–C(O)–d(ATACGTTAACGATCCTTCAC)	n/a^4^	6330.4	6330.2	15.3
***19***	*(Leu)_3_–*NH–C(O)–GGCUU^m^GAC^m^AAGUU^m^GU^m^AU^m^AU^m^GG	n/a^4^	7221.7	7222.4	9.3
***20***	*Chol**L_6_**–*NH–C(O)–GGCUU^m^GAC^m^AAGUU^m^GU^m^AU^m^AU^m^GG	n/a^4^	7394.2	7393.8	5.8
***21***	*(Leu)_3_–*NH–C(O)–***LSSL–***GGCUU^m^GAC^m^AAGUU^m^GU^m^AU^m^AU^m^GG	n/a^4^	7549.6	7551.9	3.9
***22***	*Chol**L_6_**–*NH–C(O)–***LSSL–***GGCUU^m^GAC^m^AAGUU^m^GU^m^AU^m^AU^m^GG	n/a^4^	7722.5	7722.8	4.1
***23***	*Chol**L_6_**–*NH–C(O)–***L_12_****–*NHC(O)–GGCUU^m^GAC^m^AAGUU^m^GU^m^AU^m^AU^m^GG	n/a^4^	7620.5	7619.5	2.5
***24***	*Pyr–*NH–C(O)–G^m^A^m^C^m^A^m^G^m^U^m^A^m^G^m^A^m^U^m^U^m^G^m^U^m^A^m^U^m^A^m^G^m^	n/a^4^	5972.1	5974.3	9.7
***25***	*Pyr–*NH–C(O)–***L_3_****–*NHC(O)–G^m^A^m^C^m^A^m^G^m^U^m^A^m^G^m^A^m^U^m^U^m^G^m^U^m^A^m^U^m^A^m^G^m^	n/a^4^	6074.2	6076.2	6.3

^1^ For the RP HPLC conditions, see Materials and Methods. The difference from retention time for d(TTTTTTT) (9.6 min) is given in the brackets. ^2^ Obtained by MALDI-TOF or ESI mass spectrometry. ^3^ Calculated based on molar amount of the first nucleotide attached to the polymer support (CPG). ^4^ Not available, characterized by PAGE only. ^5^ Not determined. *Chol***L_6_**–NH–, cholesteryl-6-aminohexylcarbamate residue; *Chol**L_12_***–NH–, cholesteryl-12-aminododecanylcarbamate residue; *Oleyl*–NH–, oleylamine residue; *(Leu)**_3_***–NH–, trileucine residue; *Pyr*–NH–, pyrenemethylamine residue; *Est**L_6_***–NH–, estronyl-6-aminohexylcarbamate residue; *Toc**L_6_***–NH–, α-tocopheryl-6-aminohexylcarbamate residue; *Fol**L_6_***–NH–, folate-γ-(6-aminohexylcarbamate) residue; *NH_2_**L_6_***, NH_2_–(CH_2_)_6_–; *NH_2_**L_12_***, NH_2_–(CH_2_)_12_–; ***LSSL***, –O–(CH_2_)_6_–S–S–(CH_2_)_6_–O–P(O)(OH)–; ***L_3_***, –O–(CH_2_)_3_–; ***L_12_***, –O–(CH_2_)_12_–; CH≡C–CH_2_–NH–, propargylamine residue; *Phn*, *N*-(2-hydroxyethyl)phenazinium (Supplementary Figure S2); *Cy3**L***, cyanine Cy3 with 1,2,3-triazole linker (Supplementary Figure S3); *c-B_12_*–NH–, *closo*-dodecaborane residue; N, ribonucleotide; N^m^, 2′-*O*-methylribonucleotide; d(N), deoxyribonucleotide.

## Supplementary Materials

The following are available online at https://www.mdpi.com/1420-3049/24/23/4266/s1, Figure S1: Electrophoretic analysis of reaction mixtures upon solid-phase conjugation; Figure S2: Functionalization of 5′-diamino-modified oligonucleotide (***11***) with *N*-(2-hydroxyethyl)phenazinium chloride; Figure S3: Attachment of Cy3-fluorophore to 5′-alkyne-modified oligonucleotide (***14***) using “click”-chemistry reaction; Figure S4: Representative mass spectra of the 5′-conjugates of oligonucleotides; Table S1: Amino ligands for solid-phase attachment to oligonucleotides using DSC activation and selected optimal solvents for this reaction.

## References

[1] Lönnberg H. (2009). Solid-phase synthesis of oligonucleotide conjugates useful for delivery and targeting of potential nucleic acid therapeutics. Bioconjug. Chem..

[2] Grijalvo S., Alagia A., Jorge A., Eritja R. (2018). Covalent strategies for targeting messenger and non-coding RNAs: An updated review on siRNA, miRNA and antimiR conjugates. Genes (Basel).

[3] Shevelev G.Y., Gulyak E.L., Lomzov A.A., Kuzhelev A.A., Krumkacheva O.A., Kupryushkin M.S., Tormyshev V.M., Fedin M.V., Bagryanskaya E.G., Pyshnyi D.V. (2018). A Versatile approach to attachment of triarylmethyl labels to DNA for nanoscale structural EPR studies at physiological temperatures. J. Phys. Chem. B..

[4] Zatsepin T.S., Oretskaya T.S. (2004). Synthesis and applications of oligonucleotide-carbohydrate conjugates. Chem. Biodivers..

[5] Østergaard M.E., Jackson M., Low A.E., Chappell A.G., Lee R., Peralta R.Q., Yu J., Kinberger G.A., Dan A., Carty R. (2019). Conjugation of hydrophobic moieties enhances potency of antisense oligonucleotides in the muscle of rodents and non-human primates. Nucl. Acids. Res..

[6] Astakhova I.K., Pasternak K., Campbell M.A., Gupta P., Wengel J. (2013). A locked nucleic acid-based nanocrawler: Designed and reversible movement detected by multicolor fluorescence. J. Am. Chem. Soc..

[7] Taskova M., Madsen C.S., Jensen K.J., Hansen L.H., Vester B., Astakhova K. (2017). Antisense oligonucleotides internally labeled with peptides show improved target recognition and stability to enzymatic degradation. Bioconjug. Chem..

[8] Tai W. (2019). Current aspects of siRNA bioconjugate for in vitro and in vivo delivery. Molecules.

[9] Ming X., Laing B. (2015). Bioconjugates for targeted delivery of therapeutic oligonucleotides. Adv. Drug Deliv. Rev..

[10] Jo H., Ban C. (2016). Aptamer–nanoparticle complexes as powerful diagnostic and therapeutic tools. Exp. Mol. Med..

[11] Craig K., Abrams M., Amiji M. (2018). Recent preclinical and clinical advances in oligonucleotide conjugates. Expert Opin. Drug Deliv..

[12] Takakura K., Kawamura A., Torisu Y., Koido S., Yahagi N., Saruta M. (2019). The clinical potential of oligonucleotide therapeutics against pancreatic cancer. Int. J. Mol. Sci..

[13] Capek I. (2011). Dispersions based on noble metal nanoparticles-DNA conjugates. Adv. Colloid Interface Sci..

[14] Olejniczak A., Nawrot B., Leśnikowski Z. (2018). DNA modified with boron–metal cluster complexes [M(C_2_B_9_H_11_)_2_]—synthesis, properties, and applications. Int. J. Mol. Sci..

[15] MacCulloch T., Buchberger A., Stephanopoulos N. (2019). Emerging applications of peptide–oligonucleotide conjugates: Bioactive scaffolds, self-assembling systems, and hybrid nanomaterials. Org. Biomol. Chem..

[16] Sergeeva Z.A., Lokhov S.G., Ven’yaminova A.G. (1996). Oligo(2′-O-methylribonucleotides) and their derivatives. II. Synthesis and properties of oligo(2′-O-methylribonucleotides) modified with N-(3-hydroxyethyl)phanazinium and steroid groups at the 5′-terminus. Bioorg. Khim..

[17] Iglina A.A., Meschaninova M.I., Venyaminova A.G. (2009). 5′-Lipophilic conjugates of oligonucleotides as components of cell delivery systems. Nucleic Acids Symp. Ser..

[18] Petrova N.S., Chernikov I.V., Meschaninova M.I., Dovydenko I.S., Venyaminova A.G., Zenkova M.A., Vlassov V.V., Chernolovskaya E.L. (2012). Carrier-free cellular uptake and the gene-silencing activity of the lipophilic siRNAs is strongly affected by the length of the linker between siRNA and lipophilic group. Nucleic Acids Res..

[19] Kye M., Lim Y. (2018). Synthesis and purification of self-assembling peptide-oligonucleotide conjugates by solid-phase peptide fragment condensation. J. Pept. Sci..

[20] Farzan V.M., Ulashchik E.A., Martynenko-Makaev Y.V., Kvach M.V., Aparin I.O., Brylev V.A., Prikazchikova T.A., Maklakova S.Y., Majouga A.G., Ustinov A.V. (2017). Automated solid-phase click synthesis of oligonucleotide conjugates: From small molecules to diverse N -acetylgalactosamine clusters. Bioconjug. Chem..

[21] Hermanson G. (2008). Bioconjugate Techniques.

[22] Zalipsky S., Seltzer R., Menon-Rudolph S. (1992). Evaluation of a new reagent for covalent attachment of polyethylene glycol to proteins. Biotechnol. Appl. Biochem..

[23] Wachter L., Jablonski J.A., Ramachandran K.L. (1986). A simple and efficient procedure for the synthesis of 5′-aminoalkyl oligodeoxynucleotides. Nucleic. Acids. Res..

[24] Shevelev G.Y., Krumkacheva O.A., Lomzov A.A., Kuzhelev A.A., Trukhin D.V., Rogozhnikova O.Y., Tormyshev V.M., Pyshnyi D.V., Fedin M.V., Bagryanskaya E.G. (2015). Triarylmethyl labels: Toward improving the accuracy of EPR nanoscale distance measurements in DNAs. J. Phys. Chem. B..

[25] Ballico M., Cogoi S., Drioli S., Bonora G.M. (2003). Postsynthetic conjugation of biopolymers with high molecular mass poly(ethylene glycol): Optimization of a solution process tested on synthetic oligonucleotides. Bioconjug. Chem..

[26] Vaidyanathan G., Zalutsky M.R. (1994). Improved synthesis of N-succinimidyl 4-[^18^F]fluorobenzoate and its application to the labeling of a monoclonal antibody fragment. Bioconjug. Chem..

[27] Yang M., Tsang E.M.W., Wang Y.A., Peng X., Yu H.-Z. (2005). Bioreactive surfaces prepared via the self-assembly of dendron thiols and subsequent dendrimer bridging reactions. Langmuir.

[28] Liu Z., Ren Y., Pan L., Xu H.-M. (2011). In vivo anti-tumor activity of polypeptide HM-3 modified by different polyethylene glycols (PEG). Int. J. Mol. Sci..

[29] Diala I., Osada A., Maruoka S., Imanisi T., Murao S., Ato T., Ohba H., Fujii M. (2007). Synthesis of phosphorothioate oligonucleotide–peptide conjugates by solid phase fragment condensation. Bioorg. Med. Chem. Lett..

[30] Xu Z., Peng J., Yan N., Yu H., Zhang S., Liu K., Fang Y. (2013). Simple design but marvelous performances: Molecular gels of superior strength and self-healing properties. Soft. Matter..

[31] Ghosh A.K., Doung T.T., McKee S.P., Thompson W.J. (1992). *N*,*N*′*-*dissuccinimidyl carbonate: A useful reagent for alkoxycarbonylation of amines. Tetrahedron Lett..

[32] Manoharan M., Kesavan V., Rajeev K.G. (2019). Modified iRNA agents. U.S. Patent.

[33] Trindade A.F., Frade R.F.M., Maçôas E.M.S., Graça C., Rodrigues C.A.B., Martinho J.M.G., Afonso C.A.M. (2014). “Click and go”: Simple and fast folic acid conjugation. Org. Biomol. Chem..

[34] Sivaev I.B., Semioshkin A.A., Brellochs B., Sjöberg S., Bregadze V.I. (2000). Synthesis of oxonium derivatives of the dodecahydro-closo-dodecaborate anion [B_12_H_12_]^2−^. Tetramethylene oxonium derivative of [B_12_H_12_]^2−^ as a convenient precursor for the synthesis of functional compounds for boron neutron capture therapy. Polyhedron.

[35] Semioshkin A., Nizhnik E., Godovikov I., Starikova Z., Bregadze V. (2007). Reactions of oxonium derivatives of [B_12_H_12_]^2−^ with amines: Synthesis and structure of novel B_12_-based ammonium salts and amino acids. J. Organomet. Chem..

[36] Volkov A.A., Kruglova N.S., Meschaninova M.I., Venyaminova A.G., Zenkova M.A., Vlassov V.V., Chernolovskaya E.L. (2009). Selective protection of nuclease-sensitive sites in siRNA prolongs silencing effect. Oligonucleotides.

[37] Kruglova N.S., Meschaninova M.I., Venyaminova A.G., Zenkova M.A., Vlassov V.V., Chernolovskaya E.L. (2010). Cholesterol-modified anti-MDR1 small interfering RNA: Uptake and biological activity. Mol. Biol..

[38] Ullah I., Chung K., Beloor J., Kim J., Cho M., Kim N., Lee K.Y., Kumar P., Lee S.-K. (2017). Trileucine residues in a ligand-CPP-based siRNA delivery platform improve endosomal escape of siRNA. J. Drug. Target..

[39] Singh T., Murthy A.S.N., Yang H.-J., Im J. (2018). Versatility of cell-penetrating peptides for intracellular delivery of siRNA. Drug. Deliv..

[40] Chernikov I.V., Vlassov V.V., Chernolovskaya E.L. (2019). Current development of siRNA bioconjugates: From research to the clinic. Front. Pharmacol..

[41] Chandela A., Ueno Y. (2019). Systemic delivery of small interfering RNA therapeutics: Obstacles and advances. Rev. Agric. Sci..

[42] Calabrese G., Daou A., Barbu E., Tsibouklis J. (2018). Towards carborane-functionalised structures for the treatment of brain cancer. Drug Discov. Today.

[43] Lesnikowski Z.J. (2003). Boron clusters − A new entity for DNA-oligonucleotide modification. European J. Org. Chem..

[44] Kaniowski D., Ebenryter-Olbińska K., Sobczak M., Wojtczak B., Janczak S., Leśnikowski Z., Nawrot B. (2017). High Boron-loaded DNA-oligomers as potential boron neutron capture therapy and antisense oligonucleotide dual-action anticancer agents. Molecules.

[45] Matuszewski M., Kiliszek A., Rypniewski W., Lesnikowski Z.J., Olejniczak A.B. (2015). Nucleoside bearing boron clusters and their phosphoramidites – building blocks for modified oligonucleotide synthesis. New. J. Chem..

[46] Bellon L. (2000). Oligoribonucleotides with 2′-O-(tert-Butyldimethylsilyl) Groups. Current Protocols in Nucleic Acid Chemistry.

[47] Lokhov S.G., Podyminogin M.A., Sergeev D.S., Sil’nikov V.N., Kutyavin I.V., Shishkin G.V., Zarytova V.P. (1992). Synthesis and high stability of complementary complexes of N-(2-hydroxyethyl)phenazinium derivatives of oligonucleotides. Bioconjug. Chem..

