# Supplementary material for: Novel Convenient Approach to the Solid-Phase Synthesis of Oligonucleotide Conjugates
Source: Molecules. 2019 Nov 22;24(23):4266. doi: 10.3390/molecules24234266 (PMC6930482; doi:10.3390/molecules24234266)
Supplement: Supplementary file 1 [file molecules-24-04266-s001.pdf]

Supplementary Information

Novel convenient approach to the solid-phase synthesis of oligonucleotide conjugates

Marya I. Meschaninova[1], Darya S. Novopashina[1,2,*], Olga A. Semikolenova[2], Vladimir N. Silnikov[1] and Alya G. Venyaminova[1]

[1] Institute of Chemical Biology and Fundamental Medicine SB RAS, Novosibirsk, 630090, Lavrentiev ave.8, Russia

[2] Novosibirsk State University, Novosibirsk, 630090, Pirogova str.2, Russia

* Correspondence: danov@niboch.nsc.ru; Tel.: +07-383-363-5129 (D.N.)

Amino ligands for solid-phase attachment to oligonucleotides using DSC activation and selected optimal solvents for this reaction

Table S1. The amino ligands used for solid-phase oligonucleotide conjugates synthesis.

Structure	Solvent	Molecular weight	References
**Pyrenemethylamine**<br>**(***Pyr*-NH_2**)**<br>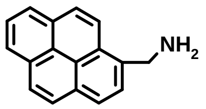	DMSO	231,29	Sigma-Aldrich
**Hexamethylenediamine**<br>**(***$NH_2$$L_6$*-$NH_2$, where ***$L_6$:*** -$(CH_2)_6$-**)**	THF	116,20	Sigma-Aldrich
**Dodecamethylenediamine**<br>**(***$NH_2$$L_{12}$*-$NH_2$, where ***$L_{12}$:*** -$(CH_2)_{12}$-**)**	THF	200,36	Sigma-Aldrich
**Aminopropanol**<br>**(*L_3*-NH_2, where *L_3:* *HO*-$(CH_2)_3$-)**	DMSO	75,11	Sigma-Aldrich
**12-Amino-1-dodecanol**<br>**(*L_{12}*-NH_2, where *L_{12}:* *HO*-$(CH_2)_{12}$-)**	DMSO	201,35	TCI Chemicals
**Propargylamine**<br>HC≡CCH_2NH_2	THF	55,08	Sigma-Aldrich
**Trileucine**<br>**(***$(Leu)_3$*-NH_2**)**<br>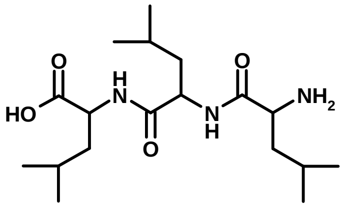	CH_3CN	357,49	Sigma-Aldrich
**Oleylamine**<br>**(***Oleyl*-NH_2**)**<br>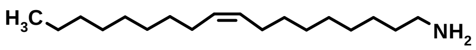	THF	267,49	Sigma-Aldrich
**Cholesteryl-6-aminohexylcarbamate (I)**<br>**(***CholL_6*-NH_2, where *L_6:* -C(O)NH$(CH_2)_6$-**)**	THF	528,85	[1]

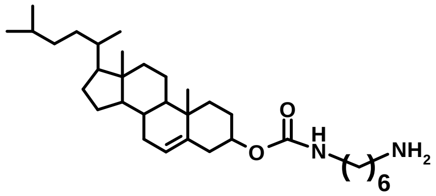			
**Cholesteryl-12-aminododecanylcarbamate (II)** (*CholL*$_{12}$-NH_2, where *L*$_{12}$: -C(O)NH(CH_2)$_{12}$-) 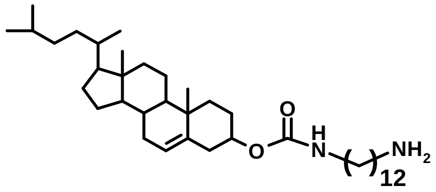	THF	613,01	by analogy with [1]
**α-Tocopheryl-6-aminohexylcarbamate (III)** (*TocL*$_6$-NH_2, where *L*$_6$: -C(O)NH(CH_2)$_6$-) 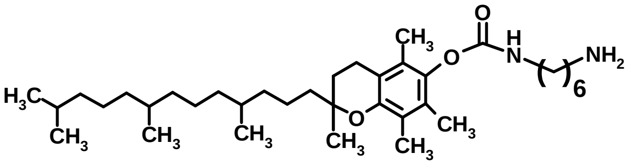	THF	572,90	by analogy with [2,3]
**Estronyl-6-aminohexylcarbamate (IV)** (*EstL*$_6$-NH_2, where *L*$_{12}$: -C(O)NH(CH_2)$_{12}$-) 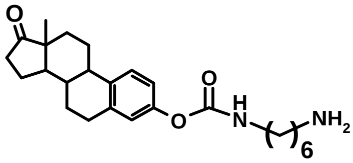	THF	412,56	by analogy with [2,3]
**Folate-γ-(6-aminohexylcarbamate) (V)** (*FolL*$_6$-NH_2, where *L*$_6$: -NH(CH_2)$_6$-) 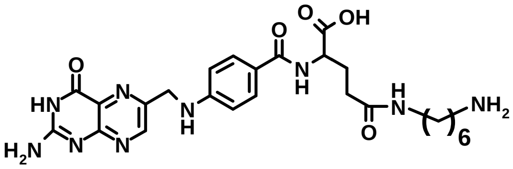	DMSO	583,60	by [4] with some modifications
**Bis-tetrabutylammonium-(4-aminobuthoxy)-undecahydro-*closo*-dodecaborate (VI)** (*c-B*$_{12}$-NH_2) 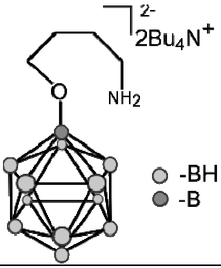	DMSO	289	[5,6]

Electrophoretic analysis of reaction mixtures upon solid-phase conjugation

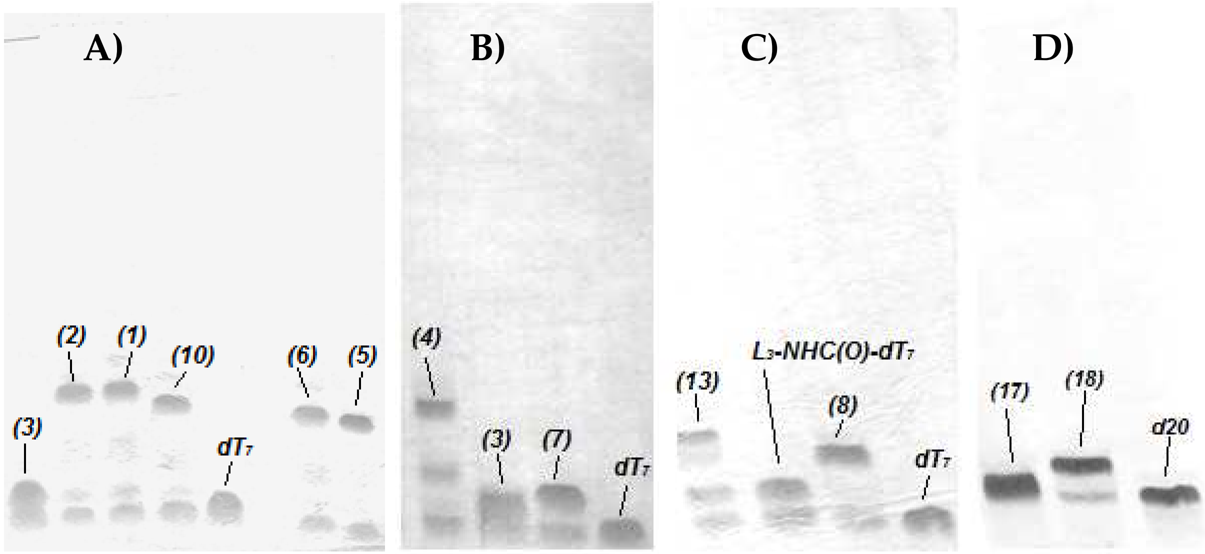

Figure S1. Analysis of reaction mixtures by PAGE: **A)** (***1***) – *Chol**L**$_6$*-NHC(O)-dT$_7$, (***2)*** – *Chol**L**$_{12}$*-NHC(O)-dT$_7$, (***3***) – *Oleyl*-NHC(O)-dT$_7$, (***5)*** – *Toc**L**$_6$*-NHC(O)-dT$_7$, ***(6)*** – *Fol**L**$_6$*-NHC(O)-dT$_7$, ***(10)*** – *NH$_2$**L**$_6$*-NHC(O)-dT$_7$; **B)** ***(3)*** – *Oleyl*-NHC(O)-dT$_7$, ***(4)*** – *Est**L**$_6$*-NHC(O)-dT$_7$, ***(7)*** – *(Leu)$_3$*-NHC(O)-dT$_7$; **C)** ***(8)*** – *Pyr*-NHC(O)-dT$_7$, ***(13)*** – *Pyr*-NHC(O)-***L**$_3$*-NHC(O)-dT$_7$; **D)** ***(17)*** – *c-B$_{12}$*-NHC(O)-d20, ***(18)*** – *Oleyl*-NHC(O)-d20. dT$_7$ = 5'-d(TTTTTTT); d20 = 5'-d(ATACGTTAACGATCCTTCAC); ***L**$_3$* = HO-(CH$_2$)$_3$-. Conditions: 15% denaturating PAAG (7M urea, acrylamide/*N*,*N*'-methylene bis-acrylamide 19:1) in TBE buffer. Gel stained with "Stains-all".

Functionalization of the 5′-diamino-modified oligonucleotides (10, 11) with N-(2-hydroxyethyl)phenazinium chloride

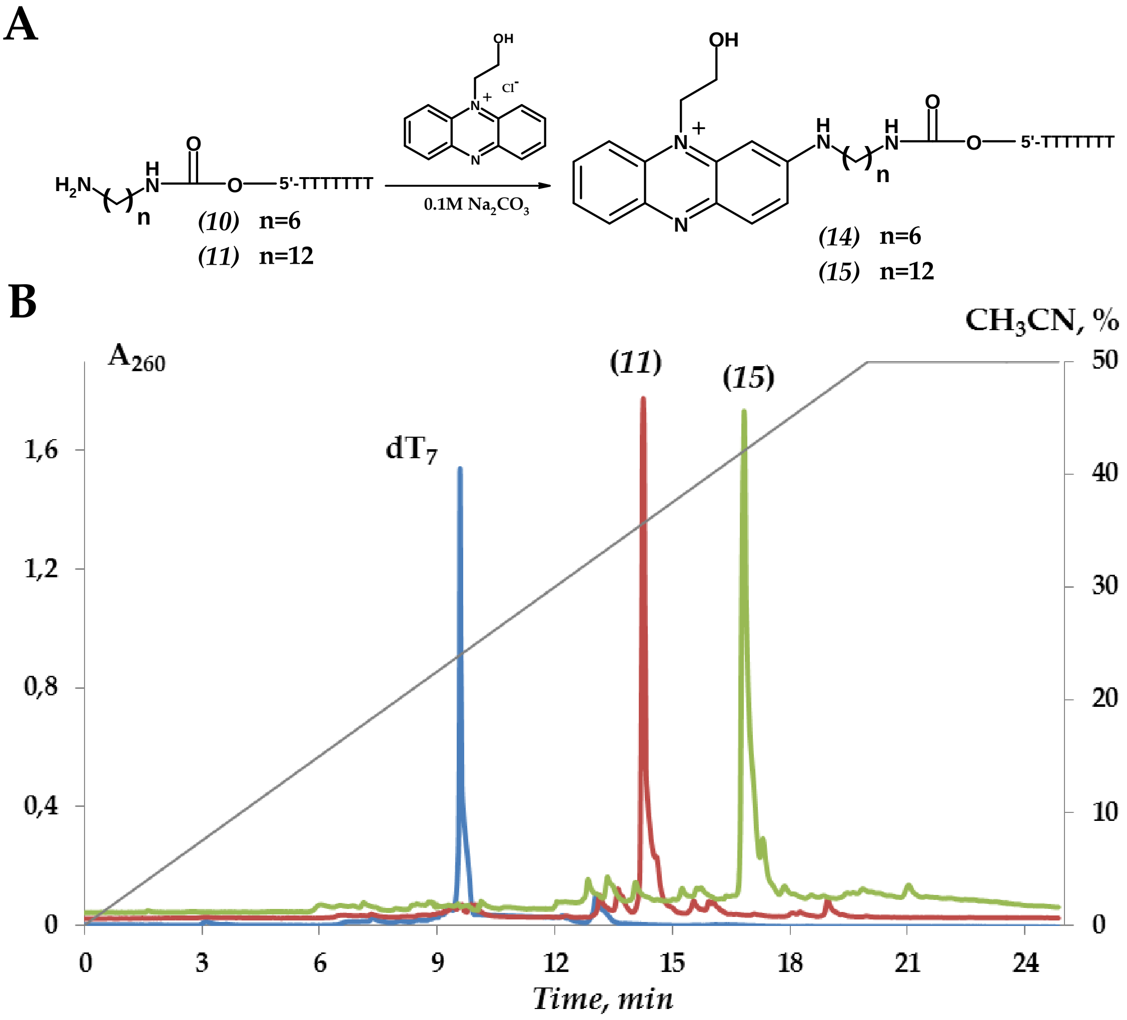

Figure S2. (**A**) Scheme of synthesis of 5'-*N*-(2-hydroxyethyl)phenazinium conjugates of dT_7 (***14, 15***) using the specific oxidation reaction of quaternary phenazinium salts of amine attachment to second position of phenazinium dye in alkaline media [7]. (**B**) RP HPLC analysis of initial oligonucleotide (dT_7) and reaction mixtures after solid-phase attachment of diamine to the activated with DSC dT_7 (*NH_2**L_{12}***-NHC(O)-dT_7 (***11***)) and *N*-(2-hydroxyethyl)phenazinium modification of diamine derivative of dT_7 in solution (*Phn-NH**L_{12}***-NHC(O)-dT_7 (***15***)).

Reverse phase-HPLC (RP-HPLC) analysis of the oligonucleotides and their conjugates was performed on Alphachrom A-02 high performance liquid chromatograph (EcoNova, Russia) with the use of ProntoSil-120-5-C18 AQ (75×2.0 mm, 5.0 µm) column, applying a gradient elution from 0 to 50% (20 min) of acetonitrile in 0.02 M triethylammonium acetate buffer, pH 7.0 at a flow rate 100 µL per min, and detection at 260 nm.

Attachment of Cy3-fluorophore to the 5′-alkyne-modified oligonucleotide (14) using "click"-chemistry reaction

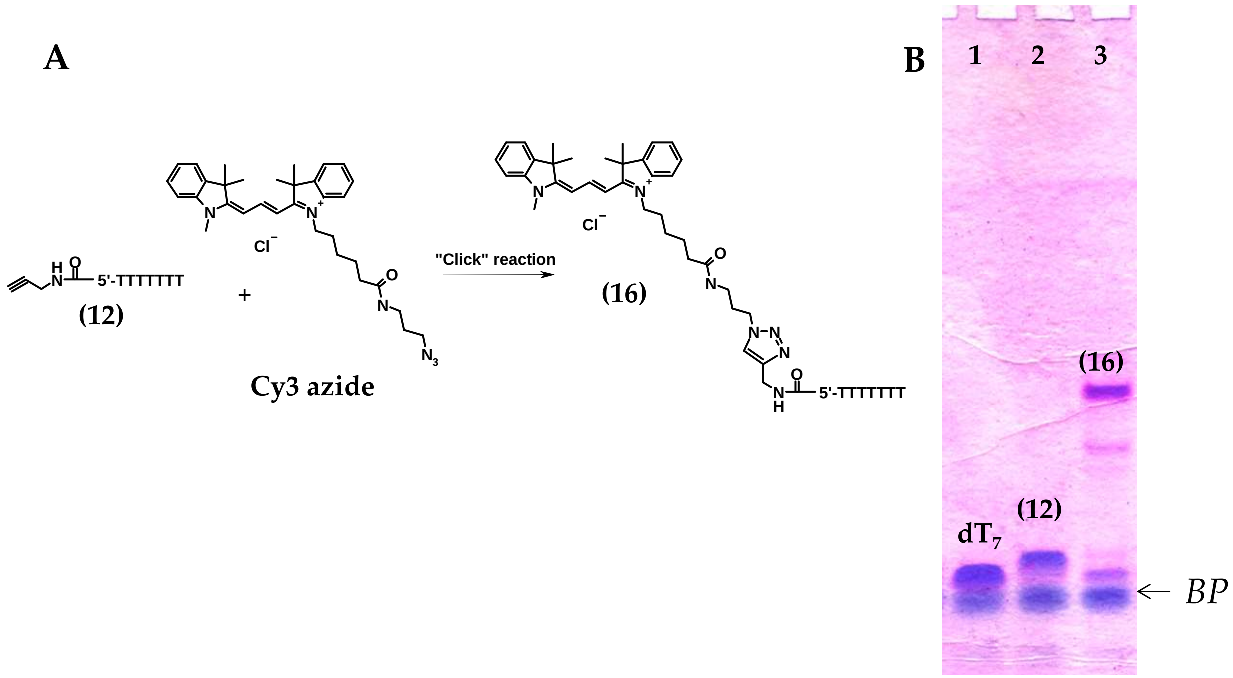

Figure S3. (**A**) Scheme of synthesis of conjugate (*16*) using "click"-reaction of Cy3 azide with 5'-propargylamine-modified dT_7 (*12*). (**B**) Analysis of reaction mixtures of dT_7 and conjugates (*12*) and (*16*) by PAGE: line **1** - deblocked reaction mixtures of initial dT_7 oligonucleotide; line **2** – deblocked reaction mixture after solid-phase attachment of propargylamine to the activated with DSC dT_7 to obtain derivative (*12*); line **3** - reaction mixture after Cy3 azide attachment to derivative (*12*) *via* "click"-chemistry in solution to obtain conjugate (*16*). Conditions: 15% denaturating PAAG (7M urea, acrylamide/*N*,*N*'-methylene bis-acrylamide 19:1) in TBE buffer. Gel stained with "Stains-all". BP – bromophenol blue.

Figure S4. ***Representative mass spectra of the 5′-conjugates of oligonucleotides***

8	*Pyr*-NH-C(O)-d(TTTTTTT)

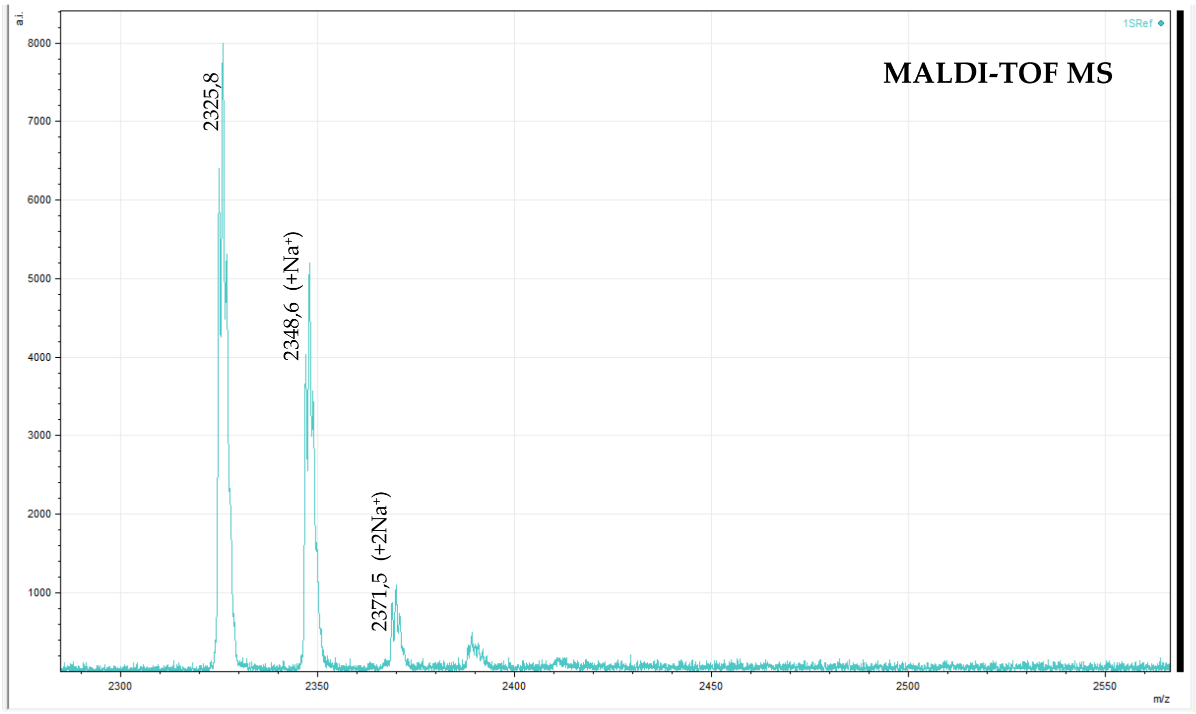

3	*Oleyl*-NH-C(O)-d(TTTTTTT)

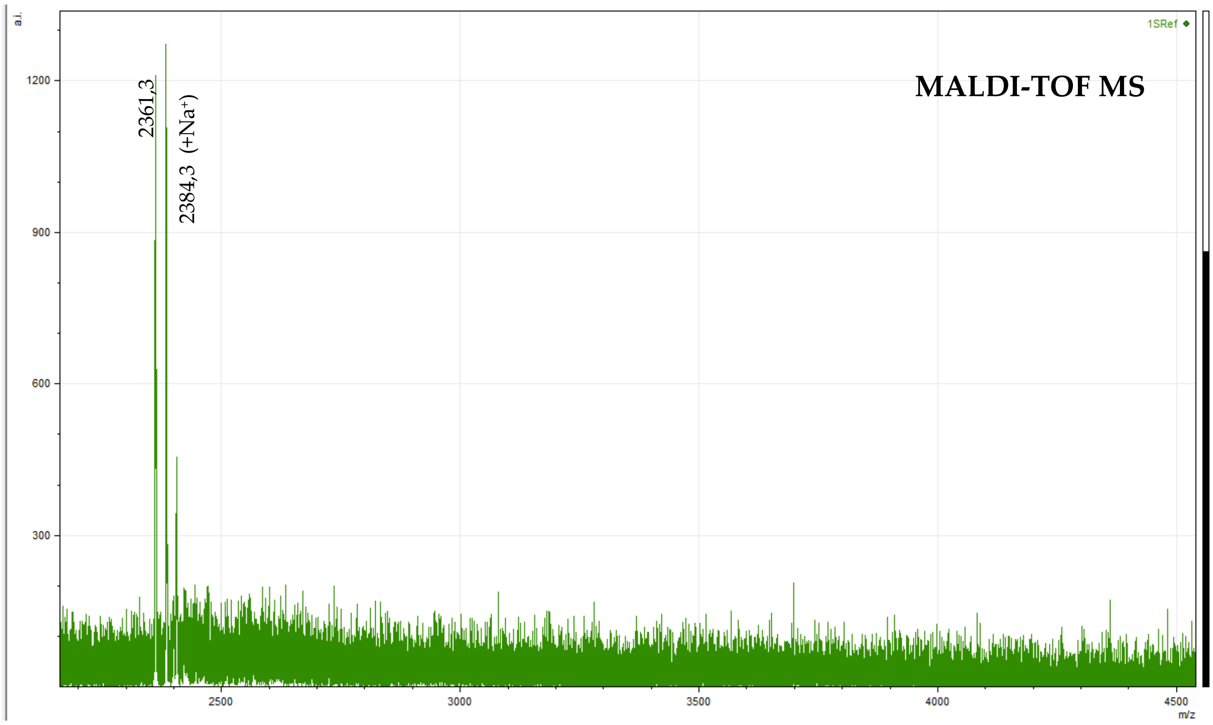

**13**	*Pyr*-NH-C(O)-***L**3*-NH-C(O)-d(TTTTTTT)

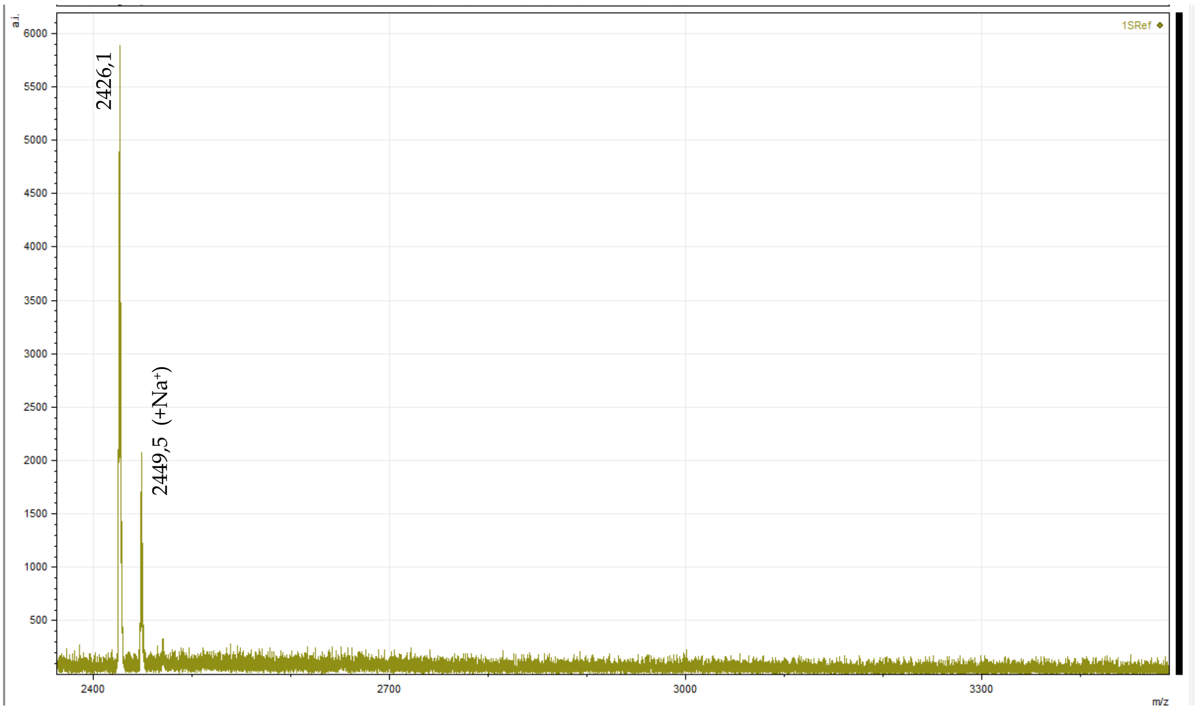

**9**	*c-B12*-NH-C(O)-d(TTTTTTT)

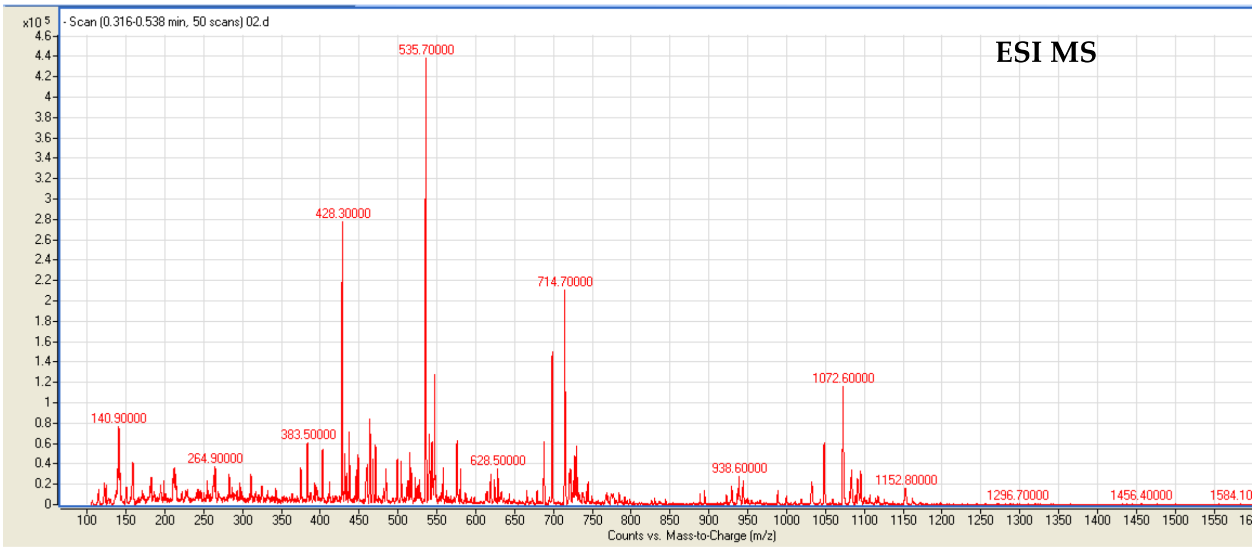

17 *c-B*$_{12}$-NH-C(O)-d(ATACGTTAACGATCCTTCAC)

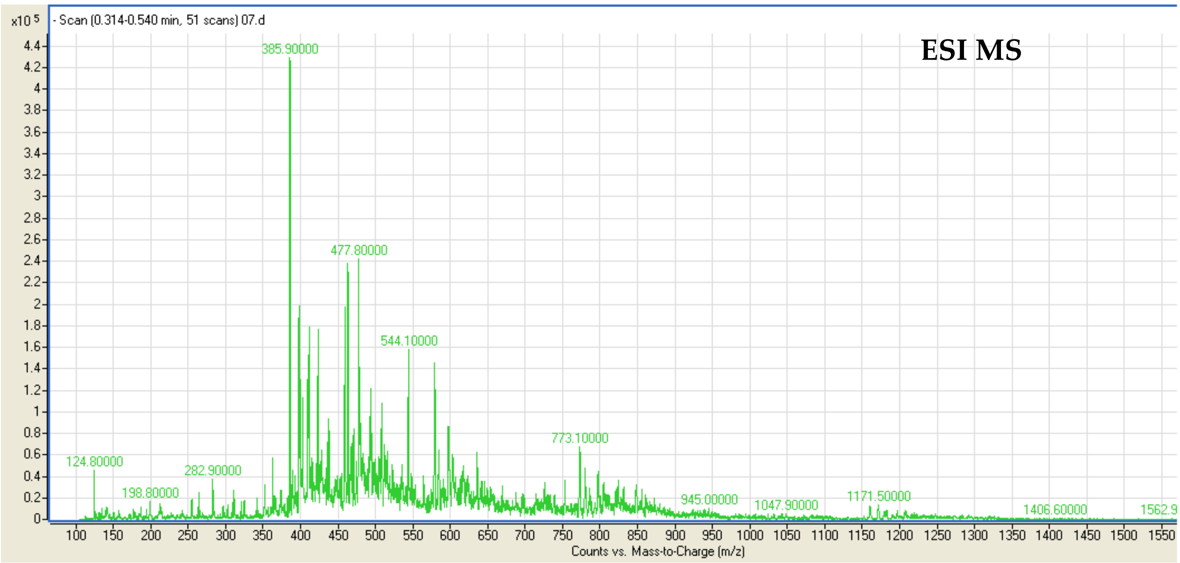

19 *(Leu)*$_{3}$-NH-C(O)-GGCUU^{m}GAC^{m}AAGUU^{m}GU^{m}AU^{m}AU^{m}GG

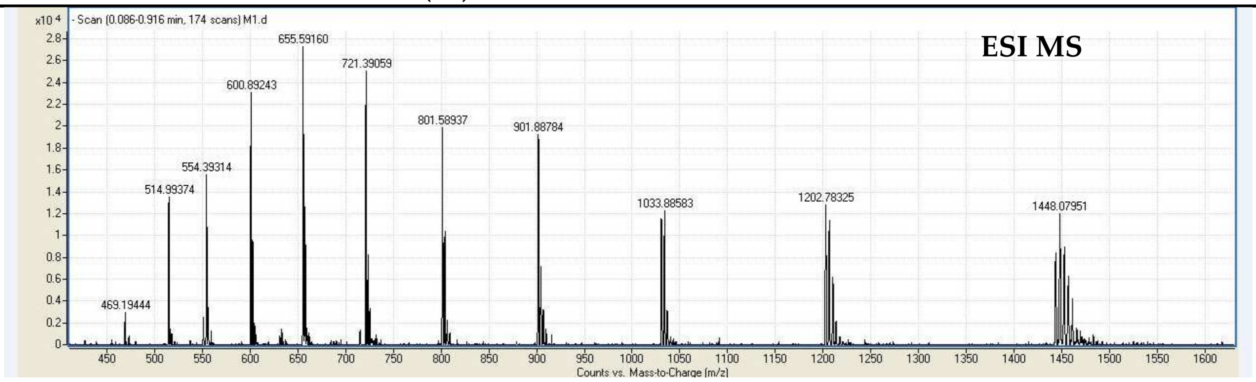

20 *Chol**L**$_{6}$*-NH-C(O)-GGCUU^{m}GAC^{m}AAGUU^{m}GU^{m}AU^{m}AU^{m}GG

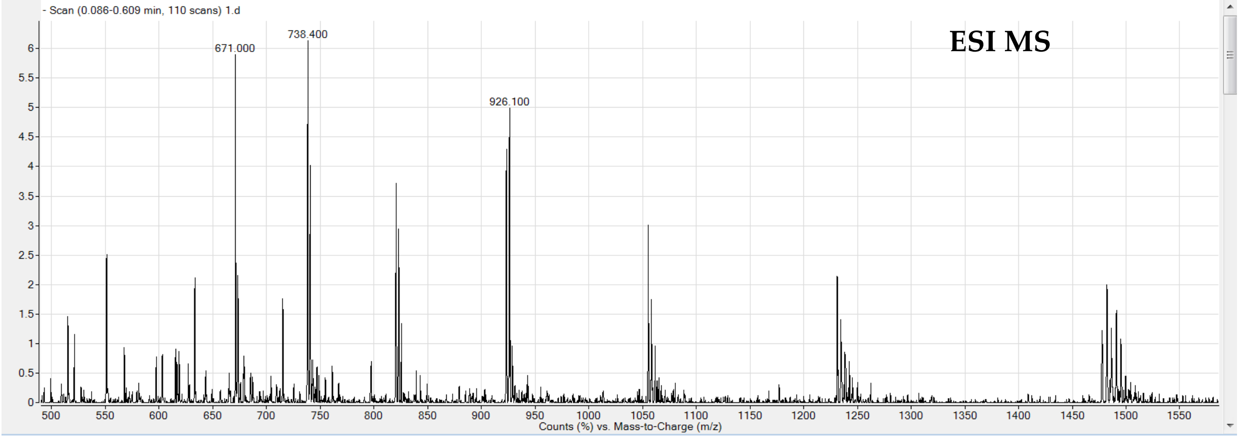

22 *Chol**L**$_6$*-NH-C(O)-***LSSL***-GGCUU^{m}GAC^{m}AAGUU^{m}GU^{m}AU^{m}AU^{m}GG

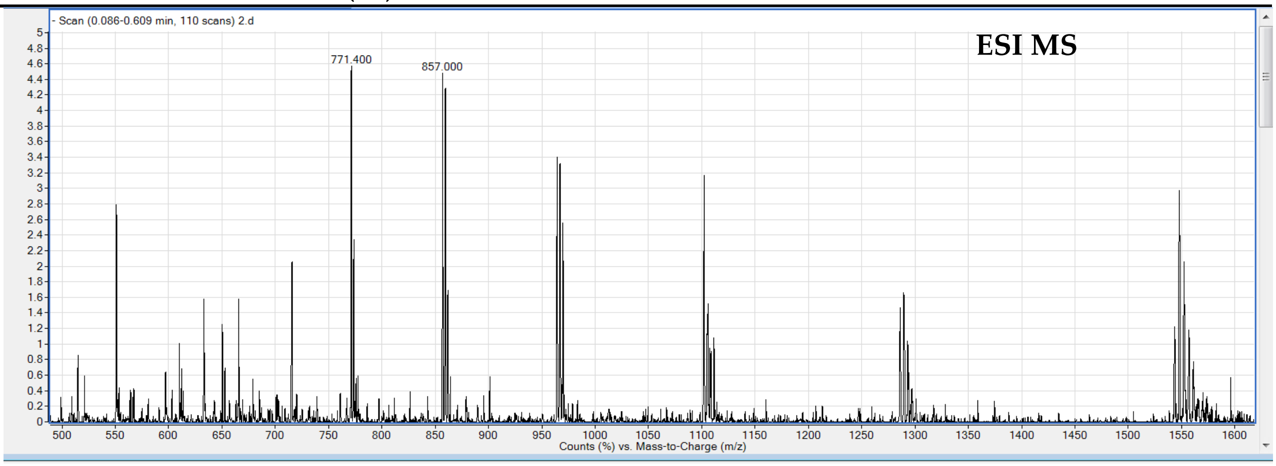

23 *Chol**L**$_6$*-NH-C(O)-***L**$_{12}$*-NHC(O)-GGCUU^{m}GAC^{m}AAGUU^{m}GU^{m}AU^{m}AU^{m}GG

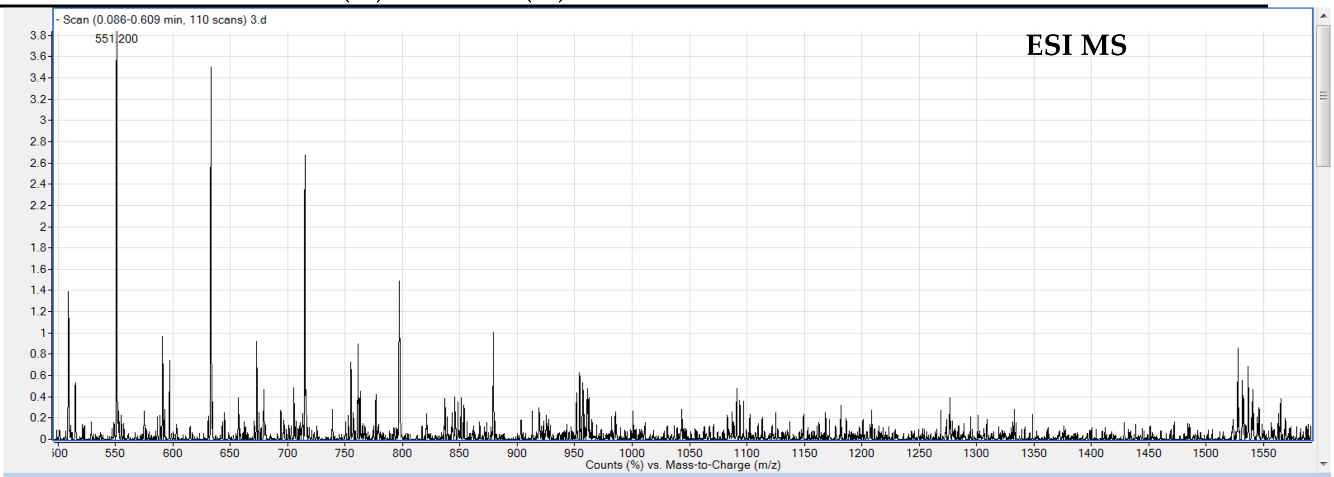

21 *(Leu)$_3$*-NH-C(O)-***LSSL***-GGCUU^{m}GAC^{m}AAGUU^{m}GU^{m}AU^{m}AU^{m}GG

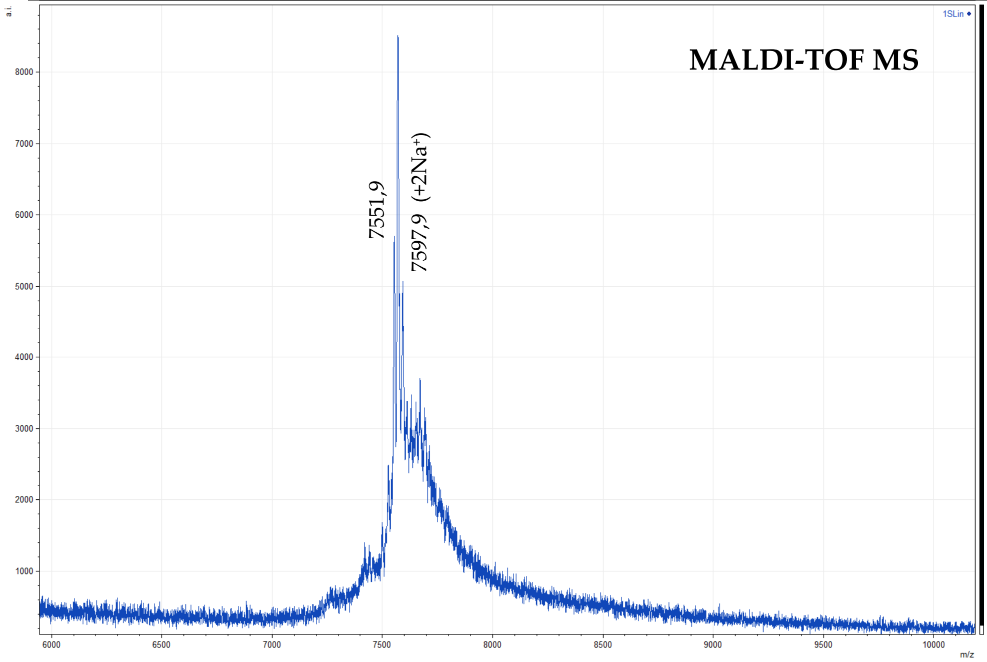

References

1. Xu, Z.; Peng, J.; Yan, N.; Yu, H.; Zhang, S.; Liu, K.; Fang, Y. Simple design but marvelous performances: molecular gels of superior strength and self-healing properties. Soft Matter. **2013**, *9*, 1091–1099; DOI:10.1039/C2SM27208C.

2. Ghosh, A. K.; Doung, T. T.; McKee, S. P.; Thompson, W. J. N,N`-Dissuccinimidyl carbonate: a useful reagent for alkoxycarbonylation of amines. Tetrahedron Lett. **1992**, *33*, 2781–2784; DOI:10.1016/S0040-4039(00)78856-3.

3. Manoharan, M., Kesavan, V., Rajeev, K. G. Modified iRNA agents. Pat. 20050107325 A1 US / Fish and Richardson Paper Corporation (U.S.) 19.05.05, U.S. Boston MA. - 245 pp.

4. Trindade, A. F.; Frade, R. F. M.; Maçôas, E. M. S.; Graça, C.; Rodrigues, C. A. B.; Martinho, J. M. G.; Afonso, C. A. M. "Click and go": simple and fast folic acid conjugation. Org. Biomol. Chem. **2014**, *12*, 3181–3190; DOI:10.1039/C4OB00150H.

5. Sivaev, I. B.; Semioshkin, A. A.; Brellochs, B.; Sjöberg, S.; Bregadze, V. I. Synthesis of oxonium derivatives of the dodecahydro-*closo*-dodecaborate anion $[B_{12}H_{12}]^{2-}$. Tetramethylene oxonium derivative of $[B_{12}H_{12}]^{2-}$ as a convenient precursor for the synthesis of functional compounds for boron neutron capture therapy. Polyhedron **2000**, *19*, 627–632; DOI:10.1016/S0277-5387(00)00293-X.

6. Semioshkin, A.; Nizhnik, E.; Godovikov, I.; Starikova, Z.; Bregadze, V. Reactions of oxonium derivatives of $[B_{12}H_{12}]^{2-}$ with amines: Synthesis and structure of novel B_{12}-based ammonium salts and amino acids. J. Organomet. Chem. **2007**, *692*, 4020–4028; DOI:10.1016/j.jorganchem.2007.06.001.

7. Lokhov, S. G.; Podyminogin, M. A.; Sergeev, D. S.; Sil'nikov, V. N.; Kutyavin, I. V.; Shishkin, G. V.; Zarytova, V. P. Synthesis and high stability of complementary complexes of N-(2-hydroxyethyl)phenazinium derivatives of oligonucleotides. *Bioconjug. Chem.* **1992**, *3*, 414–419, doi:10.1021/bc00017a010.
